# Structure confirmation, reactivity, bacterial mutagenicity and quantification of 2,2,4-tribromo-5-hydroxycyclopent-4-ene-1,3-dione in drinking water

**DOI:** 10.1038/s42004-024-01356-3

**Published:** 2024-11-14

**Authors:** Davide Ciccarelli, Ben M. J. Lancaster, D. Christopher Braddock, Matteo Calvaresi, Miroslav Mišík, Siegfried Knasmüller, Edoardo Jun Mattioli, Francesco Zerbetto, Andrew J. P. White, Tim Marczylo, Timothy W. Gant, Leon P. Barron

**Affiliations:** 1 Environmental Research Group, MRC Centre for Environment and Health, School of Public Health, Imperial College London, 86 Wood Lane, London, W12 0BZ UK; 2 NIHR-HPRU Chemical and Radiation Threats and Hazards, NIHR-HPRU Environmental Exposures and Health, MRC Centre for Environment and Health, School of Public Health, Imperial College London, 86 Wood Lane, London, W12 0BZ UK; 3 Department of Chemistry, Imperial College London, 82 Wood Lane, London, W12 0BZ UK; 4 Dipartimento di Chimica “Giacomo Ciamician”, Alma Mater Studiorum – Università di Bologna, Via Francesco Selmi 2, 40126 Bologna, Italy; 5 Medical University of Vienna, Center for Cancer Research, Borschkegasse 8a, 1090 Vienna, Austria; 6 UK Health Security Agency, Harwell Science Campus, Oxon, OX11 0RQ UK

**Keywords:** Environmental monitoring, Mass spectrometry

## Abstract

The presence of two new disinfectant by-product (DBP) groups in the UK was recently shown using non-target analysis, halogenated-hydroxycyclopentenediones and halogenated-methanesulfonic acids. In this work, we confirmed the structure of 2,2,4-tribromo-5-hydroxycyclopent-4-ene-1,3-dione (TBHCD), and quantified it together with dibromomethanesulfonic acid at 122 ± 34 and 326 ± 157 ng L^−1^ on average in London’s drinking water, respectively (*n* = 21). We found TBHCD to be photolabile and unstable in tap water and at alkaline pH. Furthermore, spectral and computational data for TBHCD and three other halogenated-hydroxycyclopentenediones indicated they could act as a source of radicals in water and in the body. Importantly, TBHCD was calculated to have a 14.5 kcal mol^−1^ lower C-Br bond dissociation enthalpy than the N-Br bond of *N*-bromosuccinimide, a common radical substitution reagent used in organic synthesis. TBHCD was mutagenic in Salmonella/microsome assays using strains TA98, TA100 and TA102. This work reveals the unique features, activity and toxicity of trihalogenated hydroxycyclopent-4-ene-1,3-diones, prompting a need to more comprehensively assess their risks.

## Abstract (web summary)

Halogenated disinfection by-products are a recognized health risk, but unequivocal identification and monitoring of new compounds is challenging, which prevents risk assessment. Here, the authors identify and quantify 2,2,4-tribromo-5-hydroxycyclopent-4-ene-1,3-dione in London drinking water, and describe the compound’s activity and toxicity.

## Introduction

A steadily increasing amount of anthropogenic chemical substances are produced worldwide^1,2^, generating an increasing potential for drinking water contamination. Environmental factors are assumed to play a major role in chronic diseases, and yet comprehensive evaluation of environmental exposures and relationship to disease is far from being achieved^3^. In fact, the number of substances analysed by target analysis is limited to a very small fraction of the substances potentially present^4^. The investigation of previously unknown potentially toxic compounds in drinking water is a long and resource-intensive process. Non-target analysis (NTA) can generate tentative identifications, but these are rarely followed-up by structure confirmation or in silico toxicityprediction^5^. For most substances, reference materials are not commercially available and synthesis is required for unequivocal structural confirmation, toxicological testing, and quantification in drinking water before risk assessment can be performed.

The health risks associated with exposure to halogenated disinfection by products (DBPs) in drinking water have been previously documented^6^. Some halogenated disinfection by-products (DBPs) are now considered probably carcinogenic to humans^7,8^. Genetic damage is a distinctive feature of human cancers^9^, and many chlorinated and brominated DBPs cause mutations in pro- and eukaryotic cells^6,10^. However, chromatographic techniques commonly used for DBP analysis, i.e., mainly gas chromatography and reversed-phase liquid chromatography (RPLC), are not necessarily suitable for very polar and ionic species, likely resulting in substantial underdetection in the past^11^. Recently, we used mixed-mode anion-exchange RPLC to conduct an in-depth investigation of acidic contaminants present in London drinking water using non-targeted and suspect screening analysis, which resulted in the discovery of emerging polar DBPs which in silico analysis flagged as potentially harmful to humans^12^. In particular, halogenated-methanesulfonic acids (HMSAs), which were previously reported and classified as DBPs in drinking water^13,14^, were subsequently confirmed to be present in London’s drinking water. These compounds were found at concentrations close to the µg L^−1^ range, and predicted as mutagenic (specifically, dibromomethanesulfonic acid)^12^. All HMSAs were also predicted as developmental toxicants by USEPA Toxicity Estimation Software Tool^12^. While predictions are useful to help with prioritization, their toxicity has to be verified. In addition to these substances, halogenated-hydroxycyclopentenediones (HHCDs) were also tentatively identified in this previous work. In particular, tribromo-HCD (TBHCD) has been previously tentatively identified, but with uncertainty about its exact structure, in Swedish and Chinese drinking water samples, as well as simulated drinking water^15–19^. TBHCD contains only one exchangeable proton and therefore requires ^13^C-NMR for detection. Gonsior et al. have recorded ^13^C NMR of complex solutions containing this compound^16^, but for the purpose of structure confirmation, a pure substance is required. Recently, it has been shown that HHCDs are present when chlorination, chloramination, ozonation/chlorination and ozonation/chloramination are employed for potabilization^20^. An in-depth analysis of the very distinctive fragmentation pattern found in the product ion scan spectrum (PIS), combined with the definition of a complete formation mechanism, allowed us to assign an unequivocal structure (2,2,4-tribromo-5-hydroxycyclopent-4-ene-1,3-dione), which was predicted as mutagenic. However, nuclear magnetic resonance (NMR) analysis and/or X-ray crystallography are necessary for unequivocal structure confirmation and have not been applied to this substance yet. Three additional tri-halogenated HCDs were also tentatively identified frequently in London drinking water and predicted as mutagenic: trichloro-HCD (TCHCD), bromodichloro-HCD (BDCHCD), and chlorodibromo-HCD (CDBHCD)^12^. Further research into HMSAs and HHCDs is required to understand their prevalence and potential health risks. As isomers might present very different toxicities, hazard evaluations based on predicted toxicity of a tentatively identified substance can be impacted by both incorrect molecular structure assignment and in silico model failure. Therefore, the following steps are fundamental in order to progress towards risk assessment of such substances: structural confirmation, development of a synthetic route for laboratory-scale preparation of pure substance, quantification in drinking water, in vitro mutagenicity assessment, reactivity and stability studies.

Our attempts to isolate TBHCD through bromination of 2,4,6-trihydroxybenzaldehyde were previously unsuccessful due to losses during evaporation^12^. Previously, TBHCD was isolated following a procedure not selective for the synthesis of TBHCD, and instead aiming at generating the compound among many other by-products. The compound was later purified by freeze-drying 1 L of combined LC fractions, and the resulting 10 mg substance were characterised by infrared spectroscopy and mass spectrometry (MS)^15^. Unfortunately, no additional structural characterisation evidence was provided (e.g., NMR or X-Ray crystallography), and the exact structure remains to be confirmed. Mass spectrometry and infrared data without additional spectroscopic measurements are not sufficient to determine with certainty which functional groups are present in the molecule and their position. With previously existing data, alternative structures like 4,5,5-tribromo-3-hydroxycyclopent-3-ene-1,2-dione could not be excluded. Until now, lack of a confirmed structure would have impacted reliable human health risk assessment. Exhaustive bromination of 4-hydroxycyclopent-4-ene-1,3-dione (HCD) has been documented, but stoichiometry, temperature, reaction concentrations and characterisation data are unavailable^21^. HCD has been previously synthesized by Claisen condensation of diethyl oxalate with diethyl-1,3-acetonedicarboxylate, followed by decarboxylation of the crude intermediate diester under acidic conditions^22^. An alternative procedure using ethyl acetoacetate with a decarboxylation-acetylation-decarboxylation protocol to avoid the need for sublimation has been reported, but insufficient detail exists to replicate it^23^. Overall, a reliable procedure for laboratory scale preparation of TBHCD is required to enable exposure and toxicological analysis.

The principal aim of this work was to investigate the occurrence, reactivity, stability and toxicity of TBHCD. The objectives included: (a) synthesis and purification of TBHCD via exhaustive bromination of 4-hydroxycyclopent-4-ene-1,3-dione; (b) TBHCD and DBMSA quantification in municipal drinking water samples to provide initial estimate of exposure; (c) In vitro mutagenicity testing of both substances using Salmonella/microsome assay with strains TA100, TA98 and TA102; (d) semi-quantification of other HHCDs and HMSAs to evaluate their relative concentration levels in municipal water; and (e) investigation of the reactivity of TBHCD. The novelty of this work lies in: (i) the first laboratory-scale synthetic preparation of TBHCD ii) the unambiguous characterisation of TBHCD by X-ray crystallography and ^13^C NMR methods; iii) the first detailed procedure in the literature for the bromination of 1,2,4-cyclopentanetrione; (iv) it is the first time that occurrence of TBHCD has been reliably quantified with quantification limits orders of magnitude lower than previous reports; (v) the first in-vitro evidence of TBHCD mutagenicity with three strains of Salmonella; and (vi) the first investigation of TBHCD reactivity and homolysis.

## Results and discussion

### Synthesis and characterisation of 2,2,4-tribromo-5-hydroxycyclopent-4-ene-1,3-dione

**Fig. 1:**
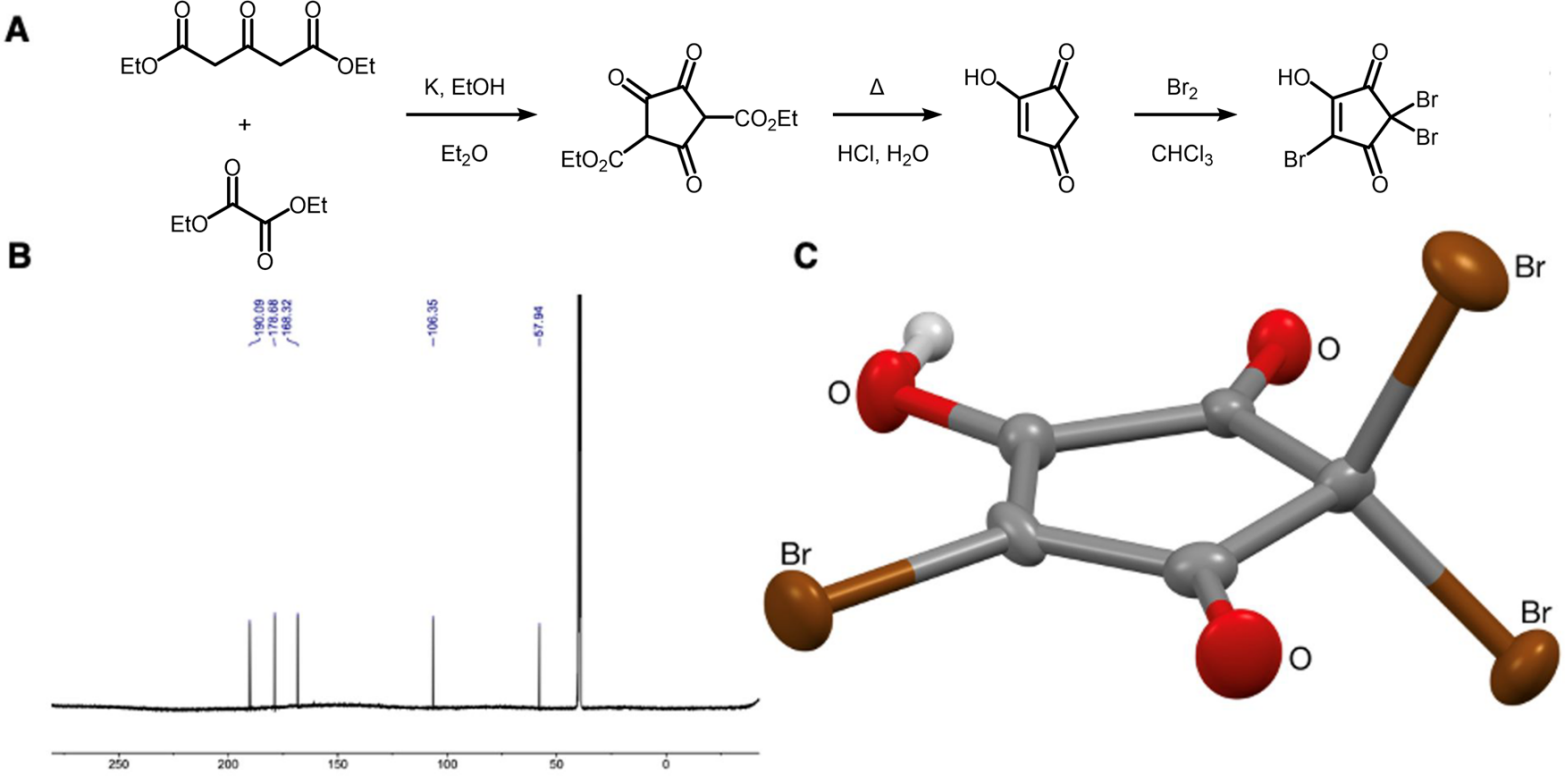
Synthesis, NMR characterisation and structural assignment of 2,2,4-tribromo-5-hydroxycyclopent-4-ene-1,3-dione. **A** Synthetic route, **B** ^13^C{^1^H} NMR inverse-gated spectrum and **C** X-ray molecular structure of 2,2,4-tribromo-5-hydroxycyclopent-4-ene-1,3-dione (50% probability ellipsoids).

As represented schematically in Fig. 1A, HCD was obtained via Claisen condensation of diethyl oxalate with diethyl-1,3-acetonedicarboxylate, followed by decarboxylation of the crude intermediate diethyl 2,4,5-trioxocyclopentane-1,3-dicarboxylate under acidic conditions. ^13^C{^1^H} NMR inverse-gated spectroscopy of the sublimed HCD indicated contamination with oxalic acid (δ_C_ = 161.0 ppm, 11%, see Figs. S2 and S3), which presumably arose from the aqueous hydrolysis of unreacted diethyl oxalate, and co-sublimes with HCD. Nonetheless, the crude HCD could be smoothly tribrominated using an excess of bromine in chloroform at 60 °C, providing pure TBHCD after concentration and recrystallisation from chloroform. The ^13^C NMR spectrum of the obtained colourless needles supported our pre-existing tentative structural assignment of TBHCD^12^ and was unambiguously confirmed by X-Ray crystallography (see Figs. 1B, C and S1)^12^. The enolic form was confirmed by the O4 longer C–O bond length, the planarity of the C5 ring, and the shortness of the C3–C4 bond compared to the rest of the C–C bonds. The mass spectrum was also consistent with previous reports^12,15^. To our knowledge, this is the first time that NMR and X-Ray chrystallography data are reported to confirm the structure of TBHCD. Characterization data were consistent with a high-purity substance.

### Analytical method development, validation and sample analysis

Considering its volatility, a large-volume direct-injection method was developed to avoid TBHCD losses during evaporation (Table S1). A mixed-mode weak-anion-exchange/reversed-phase liquid chromatography (AX-RPLC) separation enabled retention of strongly polar analytes with a pH gradient to deactivate the ion exchange functionality later in the run. This chromatographic mode increases retention capacity in comparison to RPLC methods, most likely increasing ionization efficiency when eluted in higher organic eluent compositions. In addition, separation from the void would likely improve analytical performance due to reduced matrix effects. A quadrupole time of flight mass spectrometer was operated with narrow-scan windows (0.1 Da) to maximise detector sensitivity. Method performance was evaluated according to Eurachem guidelines^24^. Excellent limits of quantification were determined at 4 and 10 ng L^−1^ for TBHCD and DBMSA with linearity verified up to 2637 and 10,560 ng L^−1^, respectively (R^2^ > 0.995, *N* ≥ 10), enabled in high resolution by large-volume injection and narrow-window acquisition. In comparison, the only previously reported limit of detection for TBHCD was 530 ng L^−1^, which would not allow for quantification in water at the concentrations found in this work^15^. Any residual chlorine in tap water matrices did not result in an obvious deviation from linearity for the standards, including at lower concentrations. Repeatability at high and low spiking level returned relative standard deviations (%RSD) of the area ≤3% for both substances (*n* = 10). Matrix effect from tap water suppressed the signal on average 1% for TBHCD and 8% for DBMSA (*n* = 10, %RSD = 2% for areas of both compounds).

**Fig. 2:**
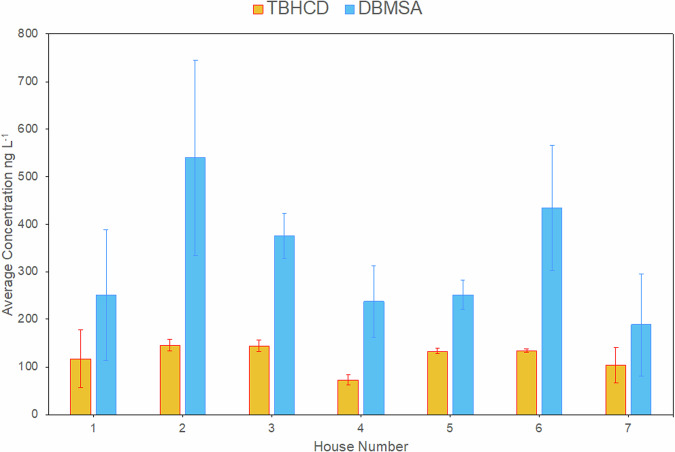
Mean concentrations of 2,2,4-tribromo-5-hydroxycyclopent-4-ene-1,3-dione (TBHCD) and dibromomethanesulfonic acid (DBMSA) in tap water from seven London households. Samples were taken in mid-August, end of August and mid-September 2023 (see also Tables S2 and S3). Error bars represent standard deviation over samples taken at three separate timepoints and not error uncertainty across repeated measurement of the same sample.

As no analyte-free tap water could be retrieved, standard addition was preferentially adopted to account for matrix effects. Calibration with a minimum of five standards returned coefficients of determination of R^2^ > 0.995 (satisfying requirements set by all available guidance)^25–28^. Municipal water from seven homes in London were sampled every two weeks for a total of six weeks (sampling coordinates and dates in Table S2, results in Table S3). Mean concentrations were 122 ± 34 and 326 ± 157 ng L^−1^ for TBHCD and DBMSA, respectively (here standard deviations represent variance occurring with sampling time and point variation, and not standard deviation for individual measurement. See Fig. 2 for details of individual sampling points).

Chlorinated HMSAs and HHCDs were semi-quantified employing DBMSA and TBHCD as reference materials, respectively, which shared structural and chromatographic retention time similarity, and in line with other works^29^. The mean semi-quantitative concentrations of DCMSA and BCMSA were 3164 ± 2097 and 1164 ± 617 ng L^−1^, in line with concentrations found in tap water samples from Europe, Asia and America^14^. BDCHCD, CDBHCD and TCHCD were semi-quantified respectively at 150 ± 35, 286 ± 75 and 138 ± 43 ng L^−1^ on average. Although monitoring only seven homes, these were spread across London and on multiple occasions. Extracted ion chromatograms for all analytes in a London tap water sample are available in Fig. S6. These results indicate potential widespread occurrence of all analytes in London municipal drinking water. Given the impossibility to account for TBHCD differential degradation due to variable residence time in the pipeline, it is not possible at this stage to analyse spatiotemporal trends. In order to do so, sampling immediately after treatment should be attempted. A more comprehensive spatiotemporal study is recommended to understand occurrence on a much larger scale.

### Stability and reactivity of TBHCD

A stability study conducted in laboratory tap water (pH = 7.8, water treatment: chlorination) showed that frozen samples were stable, but only for 24 hours (details in Table S4). Refrigerated tap water solutions showed a 12% peak area percent (A%) decrease over 24 hours and consequently could not be considered stable. Moreover, very significant transformation occurred at room temperature (RT) in tap water (85% decrease in 24 hours). However, such results were considered valid only for the drinking water composition tested. Refrigerated ultrapure water solutions were found to be stable for 24 hours. This behaviour presents particular implications for human exposure, for example, if drinking water is consumed immediately from the tap, filtered, or stored for a period of time in different containers with/without refrigeration. Whilst initially considered to be a positive outcome (i.e., the compound was transforming or degrading rapidly), further assessment of stability data revealed potentially more complex instability behaviour which could have implications for exposure. This study clearly shows that the accurate determination of TBHCD exposure on a large scale is likely very challenging. Despite variations in drinking water composition that could significantly alter the stability of TBHCD, sample freezing and analysis within 24 hours is a resource-intensive activity with obvious major logistical challenges to overcome for sampling points distant from the testing laboratory.

Odd-electron fragments in electrospray ionisation mass spectrometry are rare, mostly occurring in positive rather than negative mode, and generated by stabilization of the radical ion^30–34^. Here, all four HHCDs detected in samples shared a distinctive fragmentation pattern (see Figs. S4A, S4D, S4E and S4F), which included [M-H-Halogen^•^]^•–^, [M-H-Halogen^•^-CO]^•–^ and [C_4_HalogenO]^–^ in all recorded spectra, strongly indicating the presence of an identical structure with different halogen substituents. As the radical formation is favoured on the sp^3^-hybridised carbon, in BDCHCD [M-H-Br^•^]^•–^ it is diagnostic for the position of the bromine, making it a stereocenter. This unique fragmentation pattern highlights the existence of relatively stable dihalogenated radical species, suggesting low C-halogen bond dissociation enthalpies (BDEs) for the trihalogenated species. The PIS of dibromo-HCD (DBHCD, previously tentatively identified in drinking water)^12^ also revealed a fragment with low intensity generated by the loss of radical bromine (Br^•^)(Fig. S4B).

**Fig. 3:**
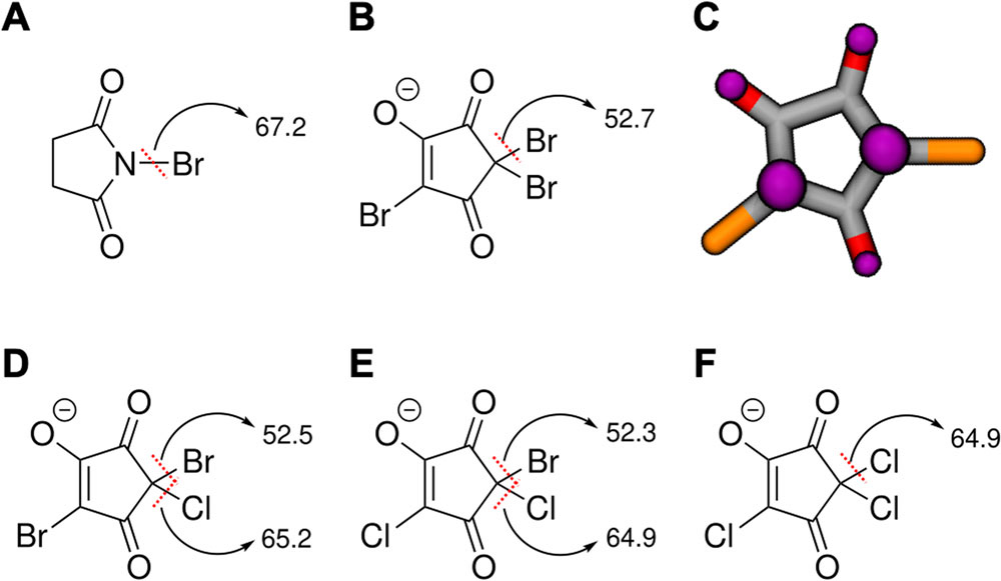
Calculation of C-Br and C-Cl homolytic cleavage BDEs (in kcal mol^-1^) for the bonds of N-bromosuccinimide. **A** 2,2,4-tribromo-5-hydroxycyclopent-4-ene-1,3-dione (TBHCD) (**B**), 2,4-dibromo-2-chloro-5-hydroxycyclopent-4-ene-1,3-dione (**D**), 2-bromo-2,4-dichloro-5-hydroxycyclopent-4-ene-1,3-dione (**E**), and 2,2,4-trichloro-5-hydroxycyclopent-4-ene-1,3-dione (**F**). Spin density distribution plot of the radical anion generated with C-Br homolysis of TBHCD is represented in **C**, spin density in purple.

**Fig. 4:**
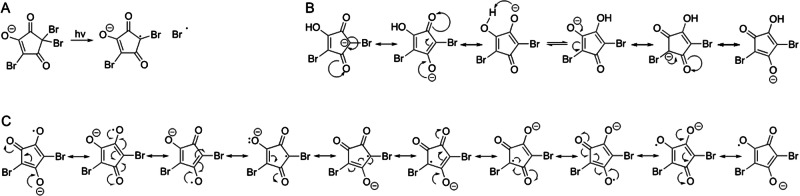
Homolysis of 2,2,4-tribromo-5-hydroxycyclopent-4-ene-1,3-dione. **A** Resonance and tautomeric structures for deprotonated 2,4-dibromo-5-hydroxycyclopent-4-ene-1,3-dione in drinking water (**B**); resonance structures for deprotonated 2,4-dibromo-5-hydroxycyclopent-4-ene-2-yl-1,3-dione (**C**). All structures are represented in anionic form reflecting ionization state at nearly-neutral pH.

Other *beta*-dicarbonyl structures like *N*-bromosuccinimide (NBS, commonly employed as an organic synthesis reagent) are a source of Br^•^^35,36^. NBS undergoes slow thermal homolysis in nitrogen atmosphere and absence of light^37^. The BDEs of NBS and of all four trihalogenated-HCDs were calculated employing a computational protocol that well reproduces the experimental BDE of NBS (BDE_calculated_=67.2 kcal mol^−1^ -Fig. 3A- vs. BDE_experimental_ = 66.0 ± kcal mol^−1^)^38^. The calculated C-Br bond BDE in TBHCD was 52.7 kcal mol^−1^ (Fig. 3B), 14.5 kcal mol^−1^ lower than that of NBS. Pronounced delocalisation of the unpaired electron (Figs. 3C and 4C) is in accordance with the favourable homolytic cleavage. C-Br BDEs were almost constant for the other brominated species: 52.5 and 52.3 kcal mol^−1^ for CDBHCD (Fig. 3D) and BDCHCD (Fig. 3E). These values are also very close to that of the hydrogen peroxide O-O bond (51.1 kcal mol^−1^)^39,40^, a well-known source of radical species in the body^41^. The calculated BDE for the C-Cl bond in TCHCD (Fig. 3F) and BDCHCD (Fig. 3E) was 64.9 kcal mol^−1^, and 65.2 kcal mol^−1^ for CDBHCD, implying that also the C-Cl bond cleavage is energetically favourable in comparison to NBS. Furthermore, in the mixed halogenated compounds, the C-Br bond is cleaved first, in agreement with the fragmentation observed in Fig. S4. Calculated bond dissociation enthalpies and free energies in the gas phase and in water are reported in Table S5.

The negative charge in all HHCDs is delocalised between oxygens in positions 3 and 5, and an identical p*K*_a_ of 4.5 was calculated for all four species with ACD/Labs Percepta software (Toronto, Ontario). Therefore, they should be present primarily in anionic form both in drinking water and blood. Figure 4B represents resonance and tautomeric structures of deprotonated DBHCD implying a symmetry axis and exchangeability of C-H hydrogen (the strongest p*K*_a_ for DBHCD was calculated at 2.4).

Standards in ultrapure water (UPW) exposed to daylight consistently exhibited degradation of TBHCD and formation of DBHCD, with a ratio between DBHCD formation and TBHCD degradation of 0.68 (*n* = 12, %RSD = 14%). When exposed to daylight at RT, the A% of TBHCD decreased by 27% of its initial area in 90 minutes on average. During the same interval, formation of DBHCD was recorded with an A% of 17% on average (details in Table S6). By comparison, reference solutions left in the dark at RT during the same interval did not show any statistically significant reduction in TBHCD or formation of DBHCD (*n* = 6). A compound tentatively identified as 4-bromo-5-hydroxycyclopent-4-ene-1,2,3-trione (BHCT) was also shown to form increasingly with daylight exposure, with a ratio between its area increase and TBHCD area decrease of 0.13 (*n* = 12, %RSD = 21%). In the absence of reference materials, A% of DBHCD and BHCT were calculated by assuming ionisation efficiencies identical to that of TBHCD. When 100 μg mL^−1^ of 2,2,6,6-tetramethylpiperidine-1-oxyl (TEMPO, a paramagnetic radical scavenger) were added to UPW, over 90 minutes of daylight exposure TBHCD degradation was decreased by an average of 92%, while DBHCD and BHCT formation was diminished by 81 and 62% on average, respectively (*n* = 6, %RSD of peak areas ≤15% for all compounds). Acetone and dimethyl sulfoxide (DMSO) are a potential source of hydrogen with a much weaker C-H bond to break in comparison to the O-H of water^38^, and therefore if the hydrogen to form DBHCD was homolytically abstracted, DBHCD formation should be favoured in these solvents. However, when TBHCD was exposed to daylight in acetone and DMSO, no statistically significant formation of DBHCD was observed (*n* = 6), while the average BHCT A% reached 4% in acetone and 22% in DMSO over 3 hours. In general, DBHCD was observed to degrade faster than TBHCD in tap water. In frozen tap water solutions, an average 47% A% drop was recorded for DBHCD in 24 hours (*n* = 3, %RSD = 3%), while no statistically significant degradation of TBHCD was observed. Therefore, degradation of DBHCD and TBHCD in drinking water is expected to occur at the same time, but at different rates, making semi-quantification of DBHCD more challenging and outside the scope of this initial work.

While no statistically significant degradation was observed at pH 4 and at a physiologically relevant pH of 7.4 during two hours at RT in the dark, at pH 8.5 and 9 the compound clearly degraded forming both DBHCD and BHCT, at a rate that increased with increasing pH (%RSD < 15% over triplicate preparations for all compounds, details in Table S7). TBHCD average A% dropped to 81% within 2 hours at pH 8.5, and to 58% within 2 hours at pH 9. Acidification of a spiked tap water sample at pH 5 did not result in improved TBHCD sample stability at RT in the dark: in 24 hours the A% decreased by over 99% on average, ruling out the possibility of prolonged sample stability by acidification. When 100 μg mL^−1^ of TEMPO were added to UPW solutions adjusted at pH 9, over 2 hours of storage in the dark at RT, TBHCD degradation decreased by an average of 70%, while DBHCD and BHCT formation also decreased by 67 and 62% on average, respectively. (*n* = 6, %RSD of the areas ≤15% for all compounds). These results clearly indicate that TBHCD degradation induced by both light and alkaline conditions involves the formation of a radical pair. Variations in homolysis rates governed by pH changes have been investigated in other cases^42–44^. Radical anions are well-known to react with water with mechanisms involving proton and electron transfer^45,46^, possibly here explaining hydrogen abstraction from a substance with strong O-H bond like water. Furthermore, a possible explanation for BHCT formation is the reaction of 2,4-dibromo-5-hydroxycyclopent-4-ene-2-yl-1,3-dione formed by homolysis with oxygen and water. As well as light-induced homolysis, light-induced heterolysis has been shown to proceed through the formation of geminate radical pairs^47^. Our results are in accordance with the notion that TEMPO promotes recombination of radical pairs via spin-exchange^48^, and scavenges free radicals with a rate is inversely proportional to the radical’s stability^49^. Therefore, the relatively stable radical pair formed by TBHCD homolysis is likely recombined in intact TBHCD via spin-exchange with TEMPO. The mechanisms of DBHCD and BHCT formation remain to be determined.

A small reduction in HHCD formation was reported in simulated drinking water by Pan et al.^15^ at pH 8.5 in comparison to pH 6.0 in presence of bromide. Our results suggest that the degradation rate is faster in alkaline conditions. Hypochlorous acid has a p*K*_a_ of 7.40, and is a much stronger oxidant in its protonated form^50^. Similarly, hypobromous acid is a much stronger oxidant in its protonated form, but has a higher p*K*_a_ of 8.55^50^. Hypochlorous acid oxidises bromide to hypobromous acid. Therefore, while at pH 6 we expect both hypochlorous and hypobromous acid to be primarily in their most oxidizing form, at pH 8.5 only a small quantity of hypobromous acid should be active. They also report an increase in HHCD formation by prolonging the contact time from 1 hour to 5 days with chlorinamination, and a decrease in HHCD formation over the same period with chlorination. However, at 5 days of exposure to chlorine, TBHCD was the only species visible. In ozonation/chlorination and ozonation/chloramination treatment, Han et al. reported decreasing intensities of HHCD with increasing ozone doses^20^. Increasing ozone doses are likely to augment the oxidation of hypobromous acid to bromate, limiting the formation of brominated HCDs. Ozone could also react with aromatic precursors of HHCDs, limiting their availability to form both chlorinated and brominated HCDs^51^. A much higher proportion of bromine HCD substitution was reported for ozonation/chlorination in comparison to ozonation/chloramination. It is important to highlight that TBHCD stability in test solutions must be taken into consideration to accurately evaluate experimental formation/degradation. The reaction pathways and kinetic of degradation of TBHCD in drinking water remain to be demonstrated as part of future research projects, requiring synthesis on a much larger scale (including radioisotopes and/or stable isotope containing test materials). Mutagenicity predictions of TBHCD isomers with structures compatible with characterization data preceding this work^15^ gave positive results (details in SI), most likely driven by the electrophilic reactivity of alpha halogenated carbonyls. However, these structures do not present the same stabilization for potential products of homolysis. To date, there is insufficient evidence to exclude that the electrophilic character of TBHCD contributes or even primarily drives its instability in treated water. To establish this, confident elucidation of DBHCD and BHCT formation mechanisms is fundamental. For routine measurement of these substances in drinking water, much more detailed stability experiments are recommended. In particular, improvement of the stability of TBHCD in solution would require a fuller understanding of their reactivity in different drinking water compositions and also the evaluation of preservatives to prevent potentially multiple reactions.

### Bacterial mutagenicity tests (Salmonella/microsome assay)

**Fig. 5:**
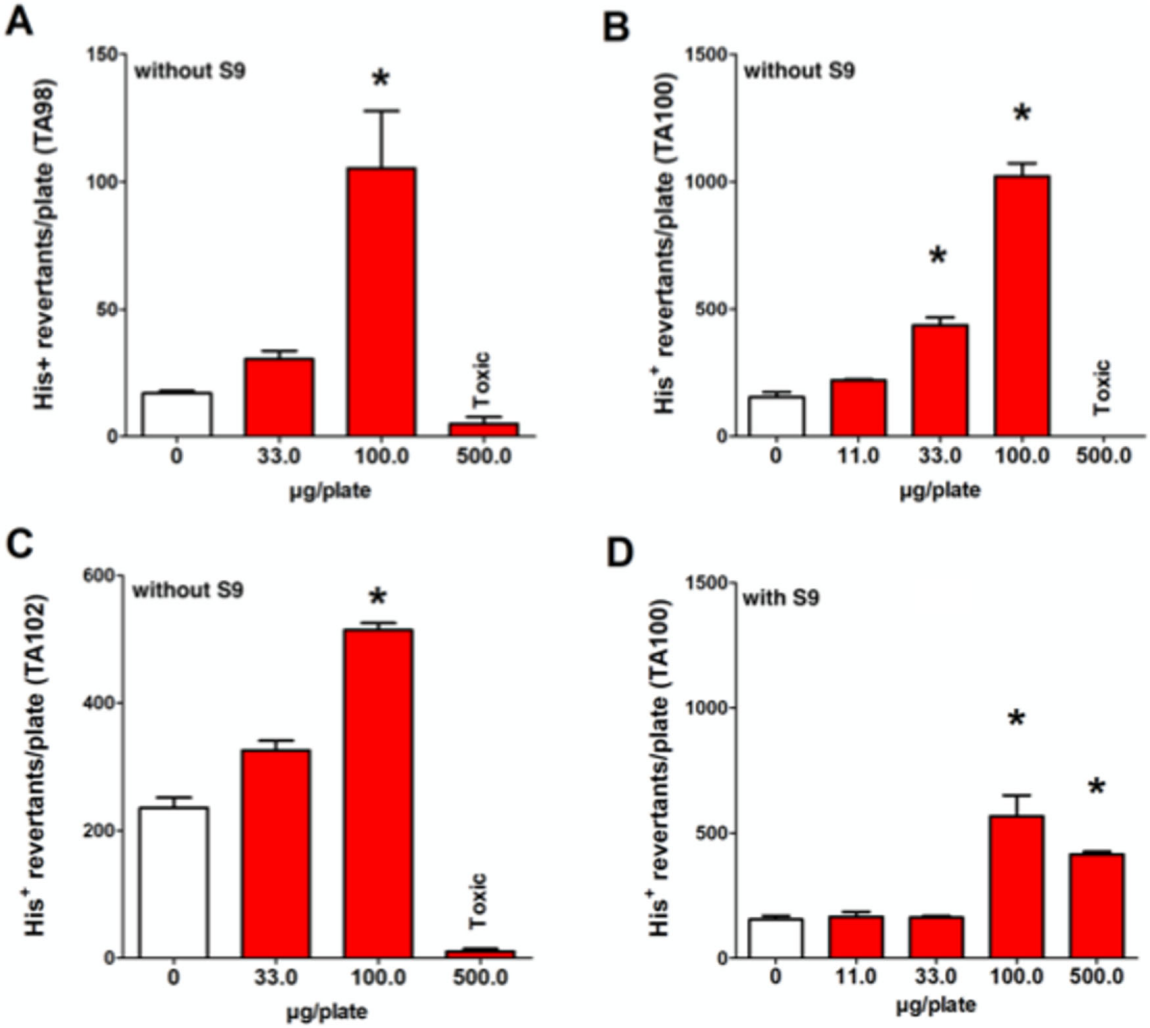
Results of a representative experiment reporting mutagenic activities 2,2,4-tribromo-5-hydroxycyclopent-4-ene-1,3-dione with Salmonella strains. **A** TA98, **B** TA100, **C** TA102 without metabolic activation, and **D** strain 100 with metabolic activation. Columns indicate mean ± SD from three plates. * positive according to the two-fold rule.

The mutagenicity of TBHCD and DBMSA was investigated with Salmonella/microsome, the most common procedure for routine mutagenicity testing of chemicals^52^, and widely employed for DBPs^6^. For this study, the following strains were employed: TA98, sensitive to frameshift mutagens^53^, TA100, base substitutions, and TA102, developed to detect genetic damage caused by radicals^54^. We found that TBHCD causes induction of His^+^ revertants at doses > 100/plate in all three tester strains (Fig. 5, individual values in Table 8A), in absence of metabolic activation mix. The highest concentration (500 µg/plate) caused acute toxicity (dissolved background lawn) which was paralleled by a decline of the revertant numbers. The most pronounced effect was detected in TA100 (6.6) followed by TA98 (6.2) and TA102 (2.2) (numbers in parenthesis indicate mutant ratios, i.e., chemically induced revertants versus background rates). Addition of metabolic activation mix (rat liver S-9) led to a decrease of the mutagenic activity by approximately 50% (Fig. 5). No evidence of mutagenic activity of DBMSA under identical conditions was detected. (See Fig. S5). Limited substance availability and TBHCD results with TA100 informed the decision to employ the remaining small amount of substance available for testing TA98 and TA102 without metabolic activation instead of experiments with an additional single strain with and without activation^55,56^. The decline of formation of His^+^ revertants after addition of the metabolic activation mix (which is added into in vitro mutagenicity tests to mimic the activation of certain indirectly-acting promutagens)^57^ was also observed in earlier experiments with halogenated DBPs^58,59^, and is probably a consequence of direct reactions of the test compound with molecules contained in the mix. However, certain brominated compounds are activated by the mix^56^.

Strains TA100 and TA98 have been employed to test the mutagenicity of other toxic DBPs, such as halogenated acetonitriles^60^, trihalomethanes and haloacetic acids^6^. Most trihalomethanes exhibited mutagenic activity only after activation with glutathione S-transferase theta. In the case of the Salmonella/microsome assay, positive results were observed primarily with a genetically modified strain RSJ100, which was not used in the present study. Most known halogenated DBPs cause base substitution mutations and are by far more active in TA100 than in other strains^10^. We found that TBHCD causes also mutations TA98 and TA102 under very similar conditions. This indicates that the compound causes DNA damage via molecular mechanisms which are different from other halogenated DBPs. The mutagenic activity of TBHCD is comparable to that observed in previous studies with other DBPs, such as dichloroacetonitrile (DCAN) and bromochloroacetonitrile (BCAN). While DCAN has a LOEC of 174 µg/plate for TA100 and 675 µg/plate forTA98 respectively, BCAN did not produce a positive response in either strains^60^. As shown in Fig. 5, our results indicate for TBHCD a LOEC of 33 µg/plate for TA100 and 100 µg/plate for TA98. For other DBPs, such as halobenzoquinones and 2-bromo-4-chloro-6-nitrophenol (2,4-BCNP), no data are yet available regarding their mutagenicity in the Salmonella microsome assay to the best of our knowledge. It must be noted that, while mutagenic activity was detected at dozens/hundreds of μg/plate, concentrations in drinking water are several orders of magnitude lower, at 122 ng L-1 on average. Obviously, no risk estimates can be inferred as this test is not quantitative, and comprehensive risk assessments should be performed in the future.

It must be noted that, at this stage, the formation of Br^•^ cannot be assumed to be responsible for the observed bacterial mutagenicity and further work is certainly required, in particular to assess the electrophilic reactivity of TBHCD with DNA. However, formation of Br^•^ is expected with light exposure and could occur in the body^41^. Hypochlorous acid is also a known mutagen^61^ and a potential source of radical chlorine with slightly higher BDE than the one here calculated for TBHCD^39^. However, hypochlorous acid quickly reacts as an electrophile in drinking water^62^. NBS, reported in this work as a reference for Br^•^ formation potential, is mutagenic in Salmonella/microsome tests (Source: European Chemicals Agency, http://echa.europa.eu/, consulted on the 30^th^ August 2023). Furthermore, NBS was shown to generate genotoxic DNA interstrand cross-links connecting 7,8-dihydro-8-oxoadenine to an opposite base^63^. Br^•^ is scavenged by dissolved organic matter (DOM) and bicarbonate, forming hydroxylated species rather than brominated ones in micropollutants degradation studies^64^, and in general reacting with antioxidant DOM by electron transfer forming bromide, rather than brominated organic species^65^. The reaction of Br^•^ with phenol, a compound representative of common DOM moieties, yields mainly *para*-benzoquinone^66^, a known mutagen^67^ and carcinogen^68,69^. Furthermore, Br^•^ is believed to be involved in the oxidation of guanine, responsible for renal carcinogenesis induced by bromate^70^.

## Conclusions

The previously unassessed mutagenicity and the unique reactivity of an emerging class of DBPs in drinking water is reported for the first time. Following successful synthesis and purification of TBHCD, ^13^C NMR and X-ray crystallography data were recorded, leading to the previously unavailable confirmation of the structure as 2,2,4-tribromo-5-hydroxycyclopent-4-ene-1,3-dione. Communicating the confidence of potentially toxic new-substance identification through appropriate combinations of suitably selective and uncorrelated techniques is critical. Many works for new DBPs are based on chromatography coupled to mass spectrometry alone. However, nuclear magnetic resonance (NMR) analysis and/or X-ray crystallography are arguably more informative for unequivocal structure confirmation and have not been applied to these substances yet. We showed that TBHCD undergoes degradation induced by both light and alkaline pH, at rates that can be significantly reduced by addition of TEMPO, clearly indicating the formation of a radical pair. Furthermore, the computationally-derived BDE for C-Br was 14.5 kcal mol^−1^ lower for TBHCD than N-Br in NBS. Chlorinated HHCDs showed also lower BDEs than NBS, clearly indicating their potential to undergo homolysis in drinking water and in the body. A much larger-scale synthesis campaign is now required to comprehensively define the reactivity of trihalogenated-HCDs in drinking water and in the human body: radiolabelling would allow identification of degradation products, as well as in vitro and in vivo metabolites. The employment of a suitable spin trapping agent for both alkyl and bromine radicals would allow direct detection of these species with electron paramagnetic resonance spectroscopy, confirming our results.

TBHCD was found to be unstable in frozen drinking water, and therefore constitutes a significant analytical challenge, as testing must be performed shortly after sample collection. Post-potabilisation conditions (residual chlorine, residence time, etc.) are therefore likely to greatly influence the concentration of TBHCD at the point-of-use. For the first time, a sensitive method for TBHCD quantification was developed, validated and applied, revealing average levels above 100 ng L^−1^ in London tap water, an intermediate concentration for a DBP. To our knowledge, this was the first time TBHCD was quantified in drinking water, which now enables a more reliable risk assessment to be conducted. CDBHCD, BDCHCD and TCHCD were semi-quantified at higher concencentrations on average. While TBHCD quantification was obtained in compliance with the results of the stability study and should therefore be considered accurate, the use of preservatives to extend sample stability could be considered in the future. However, devising reliable preservatives for this purpose requires thorough understanding of TBHCD reactivity in tap water, potentially following multiple reaction pathways. A much larger analytical campaign is now required to assess population exposure at scale.

TBHCD was shown herein to be a potent mutagen in the Salmonella/microsome assay in strains TA98, TA100 and TA102 representing a major new finding. While results of bacterial experiments cannot be employed directly for human risk assessment, genetic damage is one of the hallmarks of human cancer^9^, and therefore these observations indicate that human exposure may lead to adverse effects. TBHCD therefore exhibits the potential for toxicity via mutagenicity in line with other DBPs. The relative toxicity in comparison to these other DBPs requires further research. Importantly, the reactivity of these compounds highlights the need to understand any new chronic or acute toxicity mechanisms in humans. Now with the ability to synthesise and measure a reliable reference standard, this can progress. No mutagenic activity was detected for DBMSA. However, in consideration of predicted developmental toxicity, the widespread occurrence of DBMSA at hundreds of ng L^−1^, and the estimated concentrations of DCMSA and BCMSA at µg L^−1^ levels warrant further confirmatory toxicological investigations into these compounds.

This work is a multidisciplinary effort prompted by NTA results, where toxicity was predicted in silico for emerging chemical contaminants. Synthesis, exposure quantification and in vitro toxicity testing are resource-intensive but indispensable steps to bridge the water exposome knowledge gap and ultimately gain comprehensive control over exposure to harmful substance via drinking water.

## Methods

### Synthesis of 4-hydroxycyclopent-4-ene-1,3-dione

Potassium (2.0 g, 51 mmol) was added under nitrogen atmosphere to anhydrous ethanol (8.9 mL, 150 mmol) in diethyl ether (50 mL) at 0 °C (not stirred until complete potassium solubilisation). Diethyl oxalate (3.4 mL, 25 mmol) was added, giving a yellow homogeneous solution, followed by diethyl-1,3-acetonedicarboxylate (4.7 mL, 26 mmol) which resulted in a deep orange solution. Within minutes, the entire reaction mixture solidified. After 18 h, the solid jelly-like substance was dissolved in 2 M aqueous sulfuric acid (200 mL) and extracted with ethyl acetate (3 × 200 mL). The combined organics were dried with anhydrous magnesium sulfate, filtered, and concentrated in vacuo to yield a crude orange oil of diethyl 2,4,5-trioxocyclopentane-1,3-dicarboxylate (6.6 g), that gradually solidified and was used without further purification. Crude diester (1.3 g) was dissolved in 12 M aqueous hydrochloric acid (100 mL) and the resulting orange solution was heated to 100 °C for 90 min, during which it turned brown. The reaction mixture was allowed to cool and the solution was concentrated in vacuo to give a brown residue. This residue was then dried overnight in a vacuum desiccator at RT. Over the course of two days, the brown residue was sublimed at 120 °C under a static vacuum of 0.1 mbar to give 4-hydroxycyclopent-4-ene-1,3-dione (134 mg, 1.09 mmol, 22%) as off-white crystals contaminated with oxalic acid (11%).

mp. 117.3 − 118.5 °C (dec) (lit. 172 − 174 °C)^19^; IR (film) 1758, 1637, 1518 cm^−1^; ^1^H NMR (400 MHz, DMSO-*d*_6_) δ 12.78 (br s, oxalic acid), 6.23 (s, 1H, CH), 3.43 (br s, 1H, OH), 2.93 (s, 2H, CH_2_); ^13^C{^1^H} NMR (126 MHz, DMSO-*d*_6_) δ 198.1 (CO), 197.1 (CO), 171.6 (COH), 161.0 (oxalic acid), 120.2 (CH), 41.8 (CH_2_); HRMS (APCI + ) calcd for C_5_H_5_O_3_ (M + H)^+^: 113.0233; found: 113.0237.

### Synthesis of 2,2,4-tribromo-5-hydroxycyclopent-4-ene-1,3-dione

Bromine (0.37 mL, 7.2 mmol) was added under nitrogen atmosphere to a suspension of HCD (80.6 mg, 0.654 mmol, 11% oxalic acid) in anhydrous chloroform (2.2 mL). The resulting deep red suspension was heated to 60 °C for 2 hours and allowed to cool. The reaction mixture was concentrated in vacuo to give a pale orange solid. Recrystallisation from chloroform, employing a hot filtration to remove sparingly soluble oxalic acid, yielded colourless white crystals which were triturated with ice cold chloroform (5 × 1 mL) and dried in vacuo, providing pure 2,2,4-tribromo-5-hydroxycyclopent-4-ene-1,3-dione (95.6 mg, 42%).

mp. 190.0 − 195.3 °C (dec); IR (film) 3191, 1765, 1710, 1626 cm^−1^; ^1^H NMR (400 MHz, DMSO-*d*_6_) δ 12.38 (br s, 1H, OH); ^13^C{^1^H} NMR (126 MHz, DMSO-*d*_6_) δ 190.0 (CO), 178.8 (CO), 168.3 (CO), 106.9 (CBr), 58.2 (CBr_2_); HRMS (APCI–) calcd for C_5_^79^Br_3_O_3_ (M–H)^–^: 344.7403; found: 344.7408. Anal. Calcd for C_5_HBr_3_O_3_: C, 17.22; H, 0.29; Br, 68.74. Found: C, 17.35; H, 0.24; Br, 68.68.

### Analytical method

A large-volume (50 µL) direct-injection method was developed on a Shimadzu LCMS9030 LC-QTOF with Nexera XR LC system and standard electrospray ionisation source (Shimadzu, Kyoto, Japan). A Waters Atlantis Premier BEH C_18_ AX column (2.5 μm, 2.1 × 100 mm) was employed for gradient AX-RPLC, with a 5 mM ammonium bicarbonate solution in water (adjusted to pH 6.9 with acetic acid) as mobile phase A, and a 5 mM ammonium bicarbonate solution in acetonitrile:ultrapure water 9:1 (adjusted to pH 8.9 with diethylamine before organic solvent addition). Method performance was evaluated employing laboratory tap water (containing 132 and 560 ng L^−1^ respectively of TBHCD and DBMSA) and following procedure for sample preparation described below. Linearity was assessed in laboratory tap water spiked at 13 concentrations ranging from 10 to 10,000 ng L^−1^. Repeatability was tested in a sample spiked at 50 and 1000 ng L^−1^ (*n* = 10 for each concentration). Matrix interference was tested comparing background-corrected spiked laboratory tap water to a standard prepared in ultrapure water (solutions fortified at 500 ng L^−1^, *n* = 10). As no samples with low concentration of the analytes could be retrieved, ultrapure water spiked at 50 ng L^−1^ was employed to evaluate limits of quantification for both compounds in compliance with paragraph 6.2.2 of the Eurachem Guidelines^24^ (*n* = 10).

Samples were collected in Lightsafe 15 mL centrifuge tubes from seven household drinking water taps in London (GPS location in Table S2), frozen immediately after collection, and analysed within 24 hours. Immediately after thawing, tubes were centrifuged at 3000 rpm and 900 µL were transferred to amber glass vials. 100 µL of water:methanol 1:1 solutions containing the analytes at concentration ranging from 0 to 10000 ng L^−1^ were added, generating solutions fortified at 0, 50, 100, 200, 500 and 1000 ng L^−1^ for standard addition quantification of TBHCD and DBMSA. The amber vials were then immediately transferred to the LC autosampler at 4 °C for analysis. BDCHCD, CDBHCD and TBHCD were semi-quantified on the basis of the ratio between their peak areas and that of TBHCD in unspiked samples^29^. An identical procedure was adopted to semi-quantify DCMSA and BCMSA employing DBMSA as a reference.

### Stability and reactivity assessment

Compound stability was evaluated over 24 and 48 hours to observe potential degradation of TBHCD in municipal water samples at room temperature (20 °C), 4 °C and −18 °C, as well as in standards in ultrapure water at 4 °C. Laboratory tap water and UPW were fortified with 500 ng L^−1^ and transferred to amber LC vials, immediately stored under each condition (*n* = 3, injected in duplicate). Twenty four-hour stability at room temperature of unspiked tap water adjusted to pH 5 with acetic acid was later assessed with the same procedure. A 1000 ng L^−1^ TBHCD solution in UPW was employed to evaluate photostability of TBHCD, monitoring degradation in clear glass vials left in natural light at room temperature and sampled in duplicate at intervals of 15 minutes for 90 minutes. Reference vials in amber glass left at room temperature in the dark were sampled every 30 minutes during the same time interval (*n* = 6) and employed to evaluate degradation not ascribable to photodegradation. For all experiments in this section, the statistical significance of variations in average A% associated with a tested condition was evaluated with Student’s T-test (two tails, alpha = 0.05) in comparison to multiple injections of reference solutions (*n* ≥ 6). DBHCD and BHCT formation was approximated as A% in comparison to that of TBHCD at time zero, as their relative ionisation efficiencies could not be determined. Identical preparations containing TBHCD at 1000 ng L^−1^ with and without the addition of 100 μg mL^−1^ of TEMPO were compared after 90 minutes of exposure to natural light (*n* = 6). Duplicate 20 μg L^−1^ solutions prepared in acetone and DMSO were exposed to natural light for 1, 2 and 3 hours and compared after 20-fold dilution in water (*n* = 6 for each solvent).

To evaluate pH stability, triplicate 1000 ng L^−1^ solutions were prepared in ultrapure water adjusted to pH 4 with acetic acid, to pH 8.5 and 9 with ammonium hydroxide, and in a 10 mM phosphate buffer adjusted at pH 7.4 with ammonium hydroxide. Solutions were compared to standards in ultrapure water at the same concentration and injected after 1 and 2 hours of storage in the dark at RT (six preparations in total for each pH value). Identical preparations at pH 9 containing TBHCD at 1000 ng L^−1^, with and without the addition of 100 μg mL^−1^ of TEMPO, were compared after 2 hours of storage at RT in the dark (*n* = 6).

To calculate BDEs, geometry optimisations of the systems were carried out at the B3LYP/6-311 + + G(d,p) level of theory, using Gaussian16 (revision C.01)^71^. Frequency calculation resulted in no imaginary frequencies for all the minima confirming the nature of the critical points. BDE calculations from the optimized structures were carried out using the G3B3 procedure^72^.

### Salmonella/microsome assay

Plate incorporation assays were conducted as described in the protocol of Maron and Ames^73^, employing for both TBHCD and DBMSA strains TA98 and TA102 without metabolic activation mix (S9), and TA100 (with and without S-9). Characterisation of the strains (uvrB, rfa, pKM101 and pAQ1) was performed before the main experiments^61^. Positive and negative controls were included in all individual series. A response was considered positive when the mean number of revertant colonies was two-fold or higher the one of the negative control (i.e., the “two fold rule”)^44^. TBHCD and DBMSA were tested in plate incorporation assays and were dissolved in a mix of DMSO and water (1:2). Incubation of the plates was conducted in the dark. For each experimental point, three plates were tested in parallel. 2,4,7-trinitro-9-fluorenone (0.1 µg/plate) was used as a positive control for TA98 and caused 1061 ± 201 revertants per plate. Sodium azide (1.5 µg/plate) was used as a positive control for TA100 (without S9) and caused 636 ± 62 revertants per plate. 2-aminoantracene (2.0 µg/plate) was used in experiments employing TA100 with metabolic activation and induced 1102.0 ± 75 revertants per plate. Methyl methanesulphonate (2 µg/plate) was used as a positive control for TA102 and caused 2067 ± 153 revertants per plate.

## Supplementary information

Supplementary Information

## Data Availability

Raw data were generated at Imperial College. Derived data supporting the findings of this study are available from the corresponding author LB on request. CCDC 2307874 contains the supplementary crystallographic data for this paper. This data can be obtained free of charge via www.ccdc.cam.ac.uk/data_request/cif, or by emailing data_request@ccdc.cam.ac.uk, or by contacting The Cambridge Crystallographic Data Centre, 12 Union Road, Cambridge CB2 1EZ, UK; fax: +44 1223 336033.
