# Supplementary material for: Structure confirmation, reactivity, bacterial mutagenicity and quantification of 2,2,4-tribromo-5-hydroxycyclopent-4-ene-1,3-dione in drinking water
Source: Commun Chem. 2024 Nov 14;7:266. doi: 10.1038/s42004-024-01356-3 (PMC11564736; doi:10.1038/s42004-024-01356-3)
Supplement: Supplementary file 1 — Supplementary Information [file 42004_2024_1356_MOESM1_ESM.pdf]

Supplementary information

STRUCTURE CONFIRMATION, REACTIVITY, BACTERIAL MUTAGENICITY AND QUANTIFICATION OF 2,2,4-TRIBROMO-5-HYDROXYCYCLOPENT-4-ENE-1,3-DIONE IN DRINKING WATER

Davide Ciccarelli[a,b], Ben M. J. Lancaster[c], D. Christopher Braddock[c], Matteo Calvaresi[d], Miroslav Mišík[e], Siegfried Knasmüller[e], Edoardo Jun Mattioli[d], Francesco Zerbetto[d], Andrew J. P. White[c], Tim Marczylo[b,f], Timothy W. Gant[b,f], Leon P. Barron[a,b,f]*

[a] *Environmental Research Group, MRC Centre for Environment and Health, School of Public Health, Imperial College London, 86 Wood Lane, London W12 0BZ, UK*

[b] *NIHR-HPRU Chemical and Radiation Threats and Hazards, NIHR-HPRU Environmental Exposures and Health, MRC Centre for Environment and Health, School of Public Health, Imperial College London, 86 Wood Lane, London W12 0BZ, UK*

[c] *Department of Chemistry, Imperial College London, 82 Wood Lane, London W12 0BZ, UK*

[d] *Dipartimento di Chimica "Giacomo Ciamician", Alma Mater Studiorum – Università di Bologna, Via Francesco Selmi 2, 40126 Bologna, Italy*

[e] *Medical University of Vienna, Center for Cancer Research, Borschkegasse 8a, 1090 Vienna, Austria*

[f] *UK Health Security Agency, Harwell Science Campus, Oxon, OX11 0RQ, United Kingdom*

*Email. leon.barron@imperial.ac.uk

Summary

The materials employed for the work reported in the paper are listed below and grouped on the base of suppliers. The details of the instruments used for the characterization of the compound after synthesis are reported, as well as the details of X-ray Christal structure of 2,2,4-tribromo-5-hydroxycyclopent-4-ene-1,3-dione characterization and the cartesian coordinates of optimized structure for bond dissociation enthalpies and energies calculations. Toxicity predictions for potential isomers of 2,2,4-tribromo-5-hydroxycyclopent-4-ene-1,3-dione are reported. Supplementary figures and tables are included below.

Materials

Becton Dickinson and Company (Sparks, US): Bacto Agar.

Lancaster Synthesis (Ward Hill, USA): diethyl oxalate.

Merck (Darmstadt, Germany): Lightsafe 15 mL centrifuge tubes.

Oxoid (Basingstoke, Great Britain): Nutrient Broth II

Phenomenex (San Juan, USA): clear glass vials with polyethylene caps.

Sigma-Aldrich (Steinheim, Germany): ammonium bicarbonate, diethylamine puriss. (≥99.5%), diethyl-1,3-acetonedicarboxylate (96%), ethyl acetate and bromine (reagent grade), ammonium hydroxide >25%, sodium dihydrogen phosphate hydrate, disodium hydrogen phosphate, ammonium sodium phosphate dibasic tetrahydrate, potassium phosphate dibasic, magnesium sulphate heptahydrate, magnesium chloride hexahydrate, sodium chloride, sodium sulphate, dimethyl sulfoxide, glucose, L-histidine, D-biotin, glucose 6-phosphate, citric acid, nicotinamide adenine dinucleotide phosphate, tetracyclin, ampicilin, 2,4,7-trinitro-9-fluorenone, 2-aminoanthracene, sodium azide, methylmethansulfonate, crystal violet, 2,2,6,6-tetramethylpiperidine 1-oxyl and amber glass vials with PTFE/silicone septum caps.

Thermo Fisher Scientific (Waltham, USA): acetonitrile and acetic acid LC-MS grade, diethyl ether, trichloromethane, potassium (mineral oil, 98%), dimethyl sulfoxide.

Toronto Research Chemicals (North York, Canada): dibromomethanesulfonic acid 98%.

TRINOVA Biochem (Giessen, Germany): S9 SD rat liver homogenate.

VWR (Radnor, USA): ultrapure water and methanol LC-MS grade, ethanol, acetone, magnesium sulphate (dried), sulphuric acid 95% and hydrochloric acid 37%.

Waters (Tampa, USA): Atlantis Premier BEH C18 AX column (2.5 µm, 2.1x100mm).

Strains TA98, TA100 and TA102 were obtained as a gift from T. Grummt (German Environmental Protection Agency, Bad Elster, Germany).

Instrumentation for 2,2,4-tribromo-5-hydroxycyclopent-4-ene-1,3-dione characterization

^{1}H NMR and ^{13}C{^{1}H} NMR spectra were recorded on a Bruker AV-400 an AV-500 (Billerica, United States). ^{1}H NMR spectra were recorded at 400 MHz. ^{13}C{^{1}H} NMR spectra were recorded at 101 MHz or 126 MHz. Fourier transform infrared (IR) spectra were recorded neat using an Agilent ATR-FTIR spectrometer (Santa Clara, United States). Mass spectra were recorded by the Imperial College Department of Chemistry Mass Spectroscopy Service employing a Thermo Scientific Scientific Q-Exactive instrument. X-Ray crystallography studies were conducted by the Chemical Crystallography Laboratory in the Department of Chemistry at Imperial College London using an Agilent Xcalibur 3 E diffractometer. Melting points were recorded on an OptiMelt MPA100 (Stanford Research Systems, Sunnyvale, USA). Elemental analysis was performed in duplicate by Elemental Microanalysis Ltd, (1 Hameldown Road, Okehampton, Devon, EX20 1UB, UK), employing CE Instruments (Wigan, United Kingdom) elemental analyzer model EA1110, and a Dionex ICS1000 ion chromatograph (Sunnyvale, USA).

X-ray crystal structure of 2,2,4-tribromo-5-hydroxycyclopent-4-ene-1,3-dione

Crystal data for TBHCD: $C_5HBr_3O_3$, M = 348.79, monoclinic, $P2_1$ (no. 4), a = 6.7063(4), b = 6.9943(4), c = 8.7513(5) Å, β = 90.237(6)°, V = 410.48(4) Å^3, Z = 2, D_c = 2.822 g cm^{-3}, μ(Mo-Kα) = 14.693 mm^{-1}, T = 173 K, colourless platy needles, Agilent Xcalibur 3 E diffractometer; 1759 independent measured reflections (R_{int} = 0.0414), F^2 refinement,[X1,X2,X3] R_1(obs) = 0.0310, wR_2(all) = 0.0606, 1678 independent observed absorption-corrected reflections [$|F_o| > 4\sigma(|F_o|)$, completeness to θ_{full}(25.2°) = 99.9%], 103 parameters. The absolute structure of TBHCD was determined by use of the Flack parameter [x^+ = –0.168(19)]. CCDC 2307874.

The crystal of TBHCD that was studied was modelled as a two component twin in a *ca*. 75:25 ratio, with the two major lattices related by the twin law [1 0 0 0 –1 0 0 0 –1]. The presumed O4–H hydrogen atom could not be located from Δ*F* maps, and so it was added in an idealised position with an O–H distance of 0.90 Å and allowed to rotate about the C–O vector to find the best fit with the observed electron density (the SHELX HFIX/AFIX 147 command). The absolute structure of TBHCD was determined by use of the Flack parameter [x^+ = –0.168(19)].

References:

[X1] O.V. Dolomanov, L.J. Bourhis, R.J. Gildea, J.A.K. Howard, H. Puschmann, *J. Appl. Cryst.*, 2009, **42**, 339-341.

[X2] SHELXTL v5.1, Bruker AXS, Madison, WI, 1998.

[X3] SHELX-2013, G.M. Sheldrick, *Acta Cryst.*, 2015, **C71**, 3-8.

Toxicity prediction of potential isomers of 2,2,4-tribromo-5-hydroxycyclopent-4-ene-1,3-dione

The potential mutagenicity of three potential isomers of 2,2,4-tribromo-5-hydroxycyclopent-4-ene-1,3-dione was predicted with EPA TEST. Software, employing the nearest neighbour method. 4,5,5-tribromo-3-hydroxycyclopent-3-ene-1,2-dione, 3,5,5-tribromo-4-hydroxycyclopent-3-ene-1,2-dione, and 3,4,5-tribromo-5-hydroxycyclopent-3-ene-1,2-dione were all predicted as mutagenic.

Cartesian coordinates of optimized structure for bond dissociation enthalpies and energies calculations

----
Gas-Phase

Br
E(UB3LYP)= -2574.10577714
G3 Enthalpy= -2573.516891
G3 Free Energy= -2573.536081
Br 0.000000 0.000000 0.000000

Cl
E(UB3LYP)= -460.166882396
G3 Enthalpy= -459.990383
G3 Free Energy= -460.008421
Cl 0.000000 0.000000 0.000000

N-Bromosuccinimide
E(RB3LYP)= -2934.279525
G3 Enthalpy= -2933.370668
G3 Free Energy= -2933.411610

N	-0.099298	0.405858	0.270263
C	0.119789	-0.349781	1.435927
C	1.520223	0.005806	1.919244
C	2.066864	1.033269	0.915384
C	0.965503	1.238890	-0.117085
O	-0.659943	-1.117020	1.929992
H	2.107861	-0.913053	1.965112
H	1.441027	0.391571	2.937391

H	2.964383	0.696586	0.393075
H	2.296795	2.001160	1.364880
O	0.977506	1.959455	-1.076972
Br	-1.691426	0.310237	-0.694791

Succinimide
E(UB3LYP)= -360.072907
G3 Enthalpy= -359.746710
G3 Free Energy= -359.784122

N	-0.218803	0.681947	0.487405
C	0.121598	-0.319545	1.415684
C	1.528462	0.013143	1.934367
C	2.077215	1.041933	0.926515
C	0.943597	1.221931	-0.093952
O	-0.581792	-1.238460	1.744562
H	2.102295	-0.913849	1.980742
H	1.446870	0.402079	2.951316
H	2.967776	0.708822	0.391102
H	2.305176	2.010798	1.375394
O	1.008315	1.743942	-1.175925

2,2,4-tribromo-5-hydroxycyclopent-4-ene-1,3-dione
E(RB3LYP)= -8138.832713
G3 Enthalpy= -8136.760285
G3 Free Energy= -8136.811384

C	-0.290040	0.501774	0.099193
C	0.105800	-0.506212	1.184600
C	1.513951	-0.118117	1.676319
C	1.867968	1.024768	0.913016
C	0.902555	1.477091	-0.032415

O	-0.556795	-1.427253	1.581777
O	2.111345	-0.737338	2.548956
Br	3.530287	1.914469	1.125517
O	0.933333	2.415654	-0.813366
Br	-0.618356	-0.435374	-1.626549
Br	-1.937295	1.482768	0.635684

2,4-dibromo-5-hydroxycyclopent-4-ene-1,3-dione
E(UB3LYP)= -5564.658560
G3 Enthalpy= -5563.159446
G3 Free Energy= -5563.208855

C	-0.304662	0.733733	0.494344
C	0.044465	-0.357270	1.356396
C	1.572783	-0.173151	1.636973
C	1.941040	1.004179	0.906684
C	0.824602	1.608999	0.180479
O	-0.637190	-1.262989	1.811335
O	2.240331	-0.915993	2.340262
Br	3.684406	1.722502	0.867835
O	0.833252	2.614208	-0.526030
Br	-2.038974	1.033577	-0.182918

2,4-dibromo-2-chloro-5-hydroxycyclopent-4-ene-1,3-dione E(UB3LYP)= -6024.911648
G3 Enthalpy= -6023.253760
G3 Free Energy= -6023.303506

C	-0.299050	0.514460	0.088345
C	0.109880	-0.504917	1.162146
C	1.491965	-0.082830	1.693318
C	1.855307	1.046522	0.913271
C	0.905889	1.476109	-0.057212

O	-0.524446	-1.466866	1.505269
O	2.074002	-0.677443	2.593670
Br	3.510366	1.947772	1.139736
O	0.950145	2.388251	-0.868604
Cl	-0.695750	-0.294662	-1.475212
Br	-1.906590	1.531394	0.721200

4-bromo-2-chloro-5-hydroxycyclopent-4-ene-1,3-dione E(UB3LYP)= -3450.737407
G3 Enthalpy= -3449.653242
G3 Free Energy= -3449.701239

C	-0.293461	0.739482	0.480260
C	0.054607	-0.344826	1.350955
C	1.578207	-0.154065	1.640931
C	1.948353	1.019391	0.904908
C	0.836805	1.615595	0.168625
O	-0.633830	-1.247032	1.805226
O	2.244149	-0.891124	2.352648
Br	3.690762	1.742245	0.873356
O	0.840081	2.616618	-0.544970
Cl	-1.879784	1.005107	-0.150461

2-bromo-2,4-dichloro-5-hydroxycyclopent-4-ene-1,3-dione
E(UB3LYP)= -3910.990559
G3 Enthalpy= -3909.747097
G3 Free Energy= -3909.795450

C	-0.303935	0.511436	0.087370
C	0.104141	-0.507472	1.162345
C	1.484603	-0.088600	1.690221
C	1.848300	1.042210	0.912569
C	0.897942	1.472056	-0.056694

O	-0.534006	-1.466945	1.507448
O	2.072504	-0.680592	2.589917
Cl	3.365099	1.868537	1.119963
O	0.948147	2.387117	-0.865838
Cl	-0.700379	-0.298696	-1.476361
Br	-1.914963	1.527457	0.717059

2,4-dichloro-5-hydroxycyclopent-4-ene-1,3-dione
E(UB3LYP)=
G3 Enthalpy= -1336.146851
G3 Free Energy= -1336.193430

C	-0.301693	0.736604	0.480483
C	0.046714	-0.347520	1.351693
C	1.567710	-0.158062	1.640036
C	1.939354	1.015542	0.904984
C	0.826497	1.611316	0.169074
O	-0.642064	-1.250911	1.804812
O	2.239641	-0.891576	2.351690
Cl	3.535799	1.677186	0.877548
O	0.836983	2.613172	-0.544847
Cl	-1.888887	1.002045	-0.150114

2,2,4-trichloro-5-hydroxycyclopent-4-ene-1,3-dione
E(RB3LYP)= -1797.069377
G3 Enthalpy= -1796.240621
G3 Free Energy= -1796.287614

C	-0.296052	0.509704	0.088800
C	0.109638	-0.498925	1.179841
C	1.511390	-0.111974	1.669853
C	1.865435	1.031370	0.906106

C	0.901209	1.482930	-0.037864
O	-0.561147	-1.417482	1.571404
O	2.116550	-0.726660	2.543031
Cl	3.390487	1.847410	1.101222
O	0.932510	2.422072	-0.820091
Cl	-0.603260	-0.345883	-1.484872
Cl	-1.804009	1.399668	0.575301

Water

Br

E(UB3LYP)= -2574.10709896

G3 Enthalpy= -2573.517915

G3 Free Energy= -2573.537105

Br	0.000000	0.000000	0.000000

Cl

E(UB3LYP)= -460.168019667

G3 Enthalpy= -459.991276

G3 Free Energy= -460.009313

Cl	0.000000	0.000000	0.000000

N-Bromosuccinimide

E(RB3LYP)= -2934.293132

G3 Enthalpy= -2933.382933

G3 Free Energy= -2933.423783

N	-0.099298	0.405858	0.270263
C	0.119789	-0.349781	1.435927

C	1.520223	0.005806	1.919244
C	2.066864	1.033269	0.915384
C	0.965503	1.238890	-0.117085
O	-0.659943	-1.117020	1.929992
H	2.107861	-0.913053	1.965112
H	1.441027	0.391571	2.937391
H	2.964383	0.696586	0.393075
H	2.296795	2.001160	1.364880
O	0.977506	1.959455	-1.076972
Br	-1.691426	0.310237	-0.694791

Succinimide
E(UB3LYP)= -360.086071
G3 Enthalpy= -359.758891
G3 Free Energy= -359.796824

N	-0.220361	0.665565	0.468021
C	0.130419	-0.312602	1.419633
C	1.529934	0.010505	1.932251
C	2.076486	1.043261	0.927656
C	0.951429	1.215850	-0.088226
O	-0.597511	-1.219563	1.751831
H	2.108007	-0.914146	1.967836
H	1.455816	0.396665	2.950506
H	2.972554	0.712404	0.400670
H	2.294688	2.011431	1.382324
O	0.999250	1.743371	-1.175292

2,2,4-tribromo-5-hydroxycyclopent-4-ene-1,3-dione
E(RB3LYP)= -8138.907216

G3 Enthalpy= -8136.833954
G3 Free Energy= -8136.884931

C	-0.296738	0.502392	0.096101
C	0.105282	-0.495222	1.182309
C	1.509514	-0.114692	1.664918
C	1.870861	1.024654	0.911793
C	0.896725	1.471712	-0.024288
O	-0.553814	-1.416330	1.593030
O	2.108721	-0.743578	2.539506
Br	3.532076	1.906717	1.121242
O	0.928183	2.420583	-0.800006
Br	-0.600888	-0.441258	-1.620354
Br	-1.937170	1.477251	0.628481

2,4-dibromo-5-hydroxycyclopent-4-ene-1,3-dione
E(UB3LYP)= -5564.735934
G3 Enthalpy= -5563.235408
G3 Free Energy= -5563.284883

C	-0.309453	0.734507	0.492283
C	0.049539	-0.347651	1.352569
C	1.567640	-0.165777	1.629730
C	1.945882	1.006084	0.906310
C	0.824399	1.601213	0.186533
O	-0.627595	-1.259670	1.815762
O	2.230812	-0.918299	2.335827
Br	3.681081	1.718210	0.870270
O	0.833070	2.610994	-0.521568
Br	-2.035321	1.028186	-0.182355

2,4-dibromo-2-chloro-5-hydroxycyclopent-4-ene-1,3-dione

E(RB3LYP)= -6024.986092
G3 Enthalpy= -6023.327575
G3 Free Energy= -6023.377076

C	-0.305215	0.515554	0.086800
C	0.111197	-0.496989	1.157419
C	1.486849	-0.078416	1.683232
C	1.857556	1.047530	0.913575
C	0.902167	1.468358	-0.052258
O	-0.517564	-1.461615	1.511188
O	2.068518	-0.680264	2.588401
Br	3.507225	1.946253	1.142517
O	0.950076	2.385166	-0.864687
Cl	-0.690591	-0.296785	-1.472705
Br	-1.898498	1.528996	0.722448

4-bromo-2-chloro-5-hydroxycyclopent-4-ene-1,3-dione
E(UB3LYP)= -3450.814951
G3 Enthalpy= -3449.729900
G3 Free Energy= -3449.777563

C	-0.293230	0.734799	0.484138
C	0.064012	-0.340880	1.354057
C	1.578101	-0.153806	1.638870
C	1.956851	1.014489	0.910915
C	0.838781	1.602215	0.180302
O	-0.614118	-1.251679	1.818805
O	2.244530	-0.897579	2.352269
Cl	3.545255	1.673001	0.883171
O	0.855529	2.608289	-0.533491
Br	-2.015656	1.018947	-0.203677

2-bromo-2,4-dichloro-5-hydroxycyclopent-4-ene-1,3-dione
E(RB3LYP)= -3911.058619
G3 Enthalpy= -3909.821339
G3 Free Energy= -3909.869501

C	-0.296052	0.509704	0.088800
C	0.109638	-0.498925	1.179841
C	1.511390	-0.111974	1.669853
C	1.865435	1.031370	0.906106
C	0.901209	1.482930	-0.037864
O	-0.561147	-1.417482	1.571404
O	2.116550	-0.726660	2.543031
Cl	3.390487	1.847410	1.101222
O	0.932510	2.422072	-0.820091
Cl	-0.603260	-0.345883	-1.484872
Br	-2.099307	1.573947	0.670571

2,4-dichloro-5-hydroxycyclopent-4-ene-1,3-dione
E(UB3LYP)= -1336.893953
G3 Enthalpy= -1336.223679
G3 Free Energy= -1336.270152

C	-0.305319	0.736923	0.478913
C	0.051330	-0.339339	1.347266
C	1.563336	-0.151406	1.633553
C	1.943118	1.017032	0.905332
C	0.826710	1.603393	0.173685
O	-0.632442	-1.247957	1.809457
O	2.229562	-0.895073	2.347563
Cl	3.531611	1.675953	0.879774
O	0.837121	2.609032	-0.541582
Cl	-1.884973	0.999238	-0.148604

2,2,4-trichloro-5-hydroxycyclopent-4-ene-1,3-dione
E(RB3LYP)= -1797.144113
G3 Enthalpy= -1796.314349
G3 Free Energy= -1796.361352

C	-0.300685	0.509438	0.086563
C	0.109719	-0.490685	1.177547
C	1.506899	-0.108384	1.660687
C	1.867261	1.031323	0.906164
C	0.897712	1.475776	-0.032684
O	-0.555255	-1.411744	1.578497
O	2.113079	-0.731222	2.536275
Cl	3.387730	1.845324	1.100484
O	0.930880	2.421879	-0.812403
Cl	-0.594273	-0.348528	-1.480268
Cl	-1.800314	1.399052	0.571869

Supplementary figures

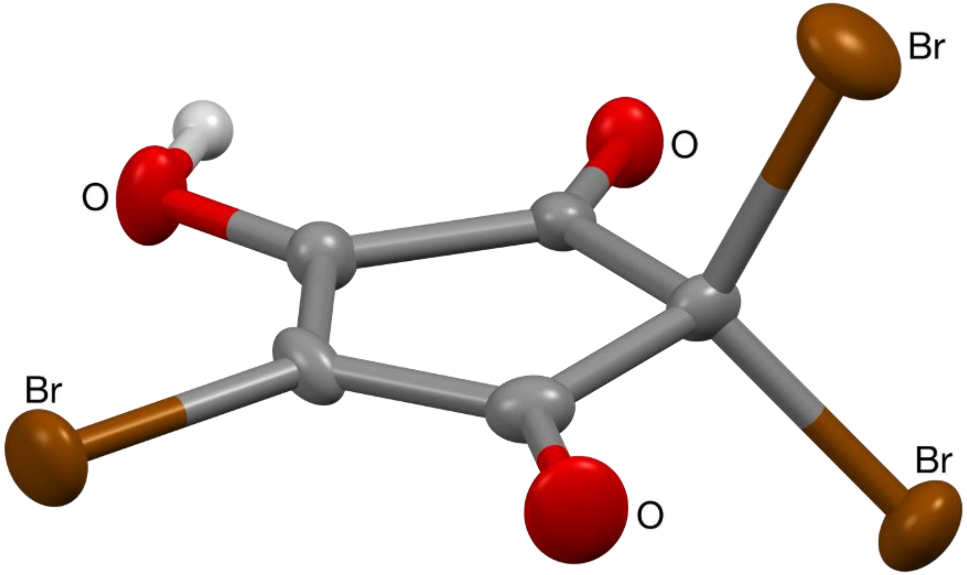

Figure S1: The crystal structure of TBHCD (50% probability ellipsoids).

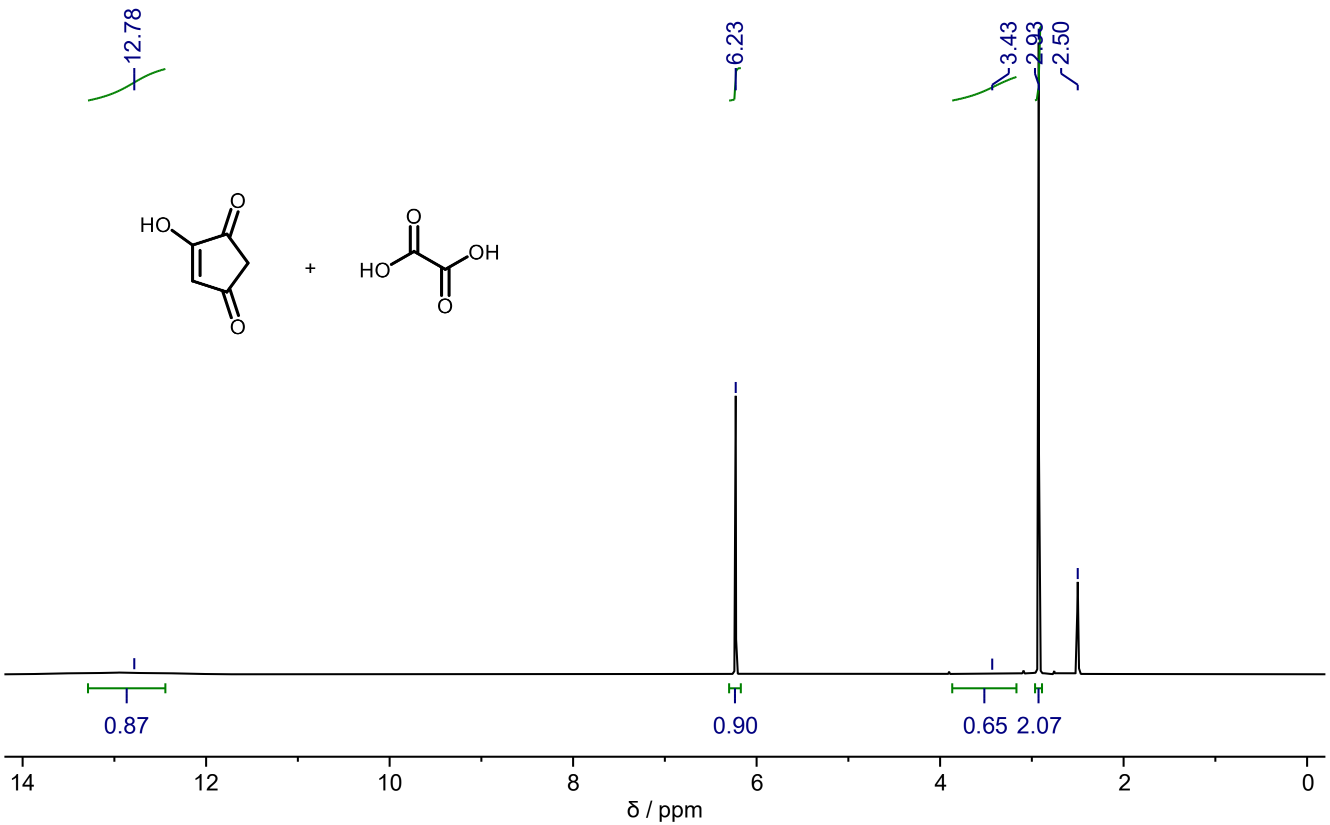

Figure S2: ^{1}H NMR spectrum of oxalic acid contaminated 4-hydroxycyclopent-4-ene-1,3-dione (**2**) (400 MHz, DMSO-d_6)

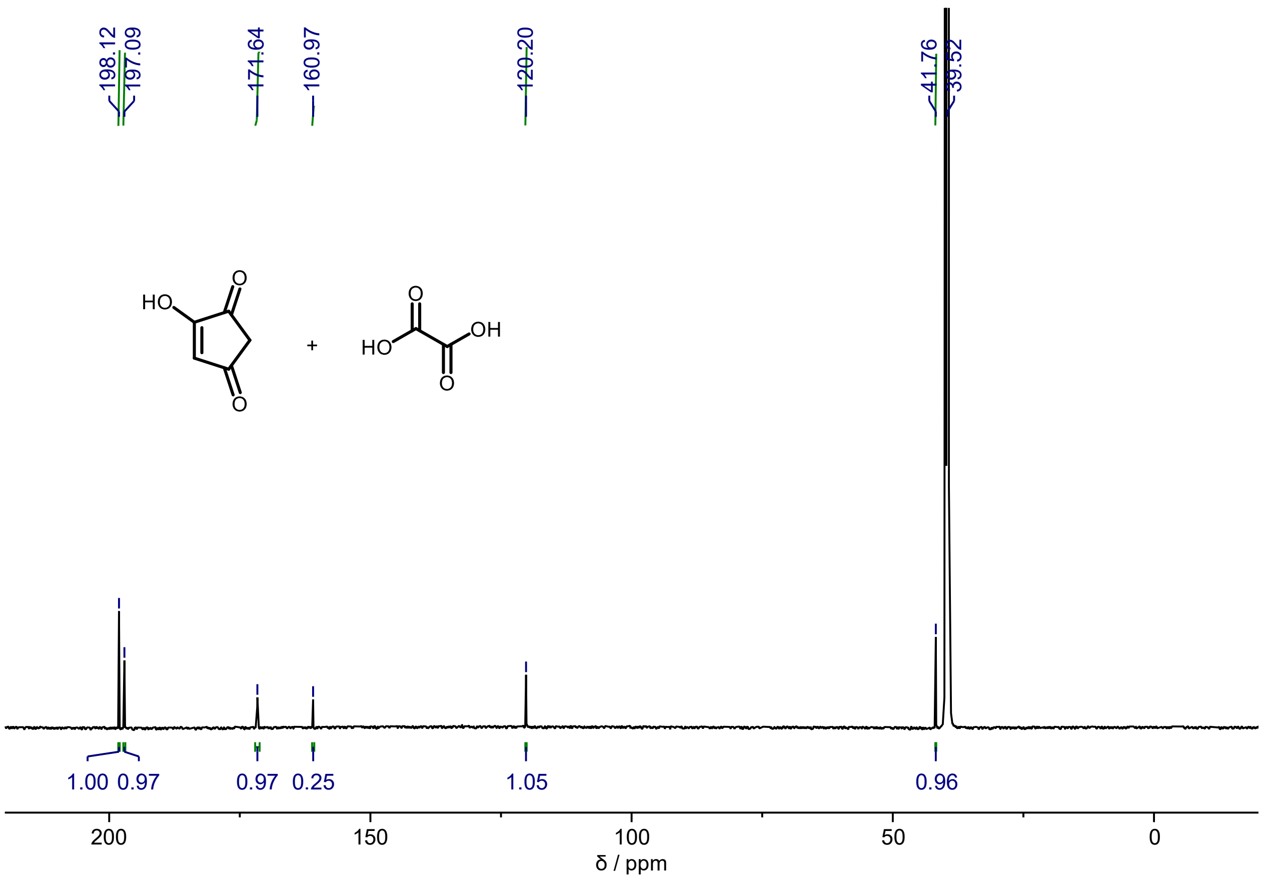

Figure S3: $^{13}C\{^{1}H\}$ NMR inverse-gated spectrum of oxalic acid contaminated 4-hydroxycyclopent-4-ene-1,3-dione (2) (126 MHz, DMSO-d6)

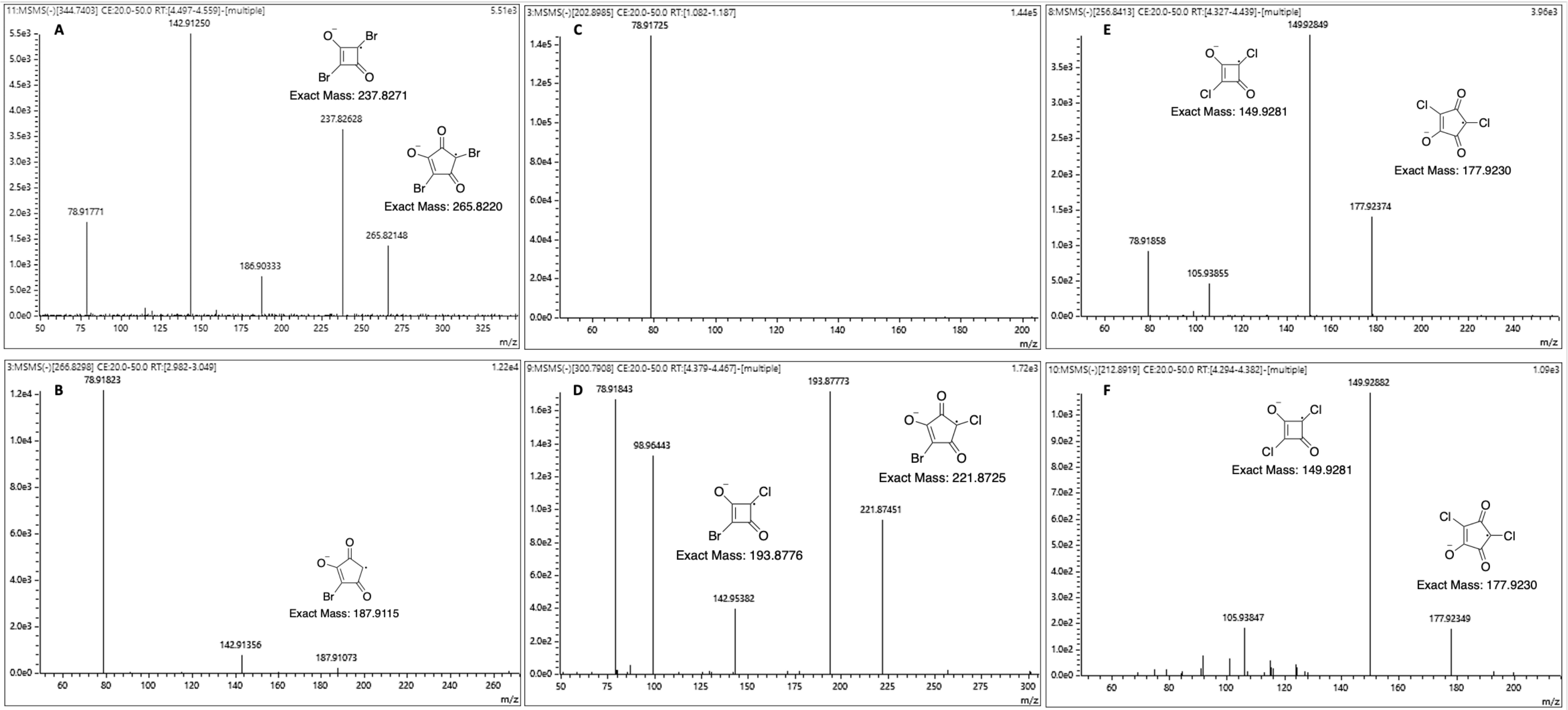

Figure S4. Product ion scan spectra of 2,2,4-tribromo-5-hydroxycyclopent-4-ene-1,3-dione (A), 2,4-dibromo-5-hydroxycyclopent-4-ene-1,3-dione (B), 4-bromo-5-hydroxycyclopent-4-ene-1,2,3-trione (C), 2,4-dibromo-2-chloro or 2,2-dibromo-4-chloro-5-hydroxycyclopent-4-ene-1,3-dione (D), 2-bromo-2,4-dichloro-5-hydroxycyclopent-4-ene-1,3-dione (E) and 2,2,4-trichloro-5-hydroxycyclopent-4-ene-1,3-dione (F), with structures and exact masses of odd-electrons species present in the spectra.

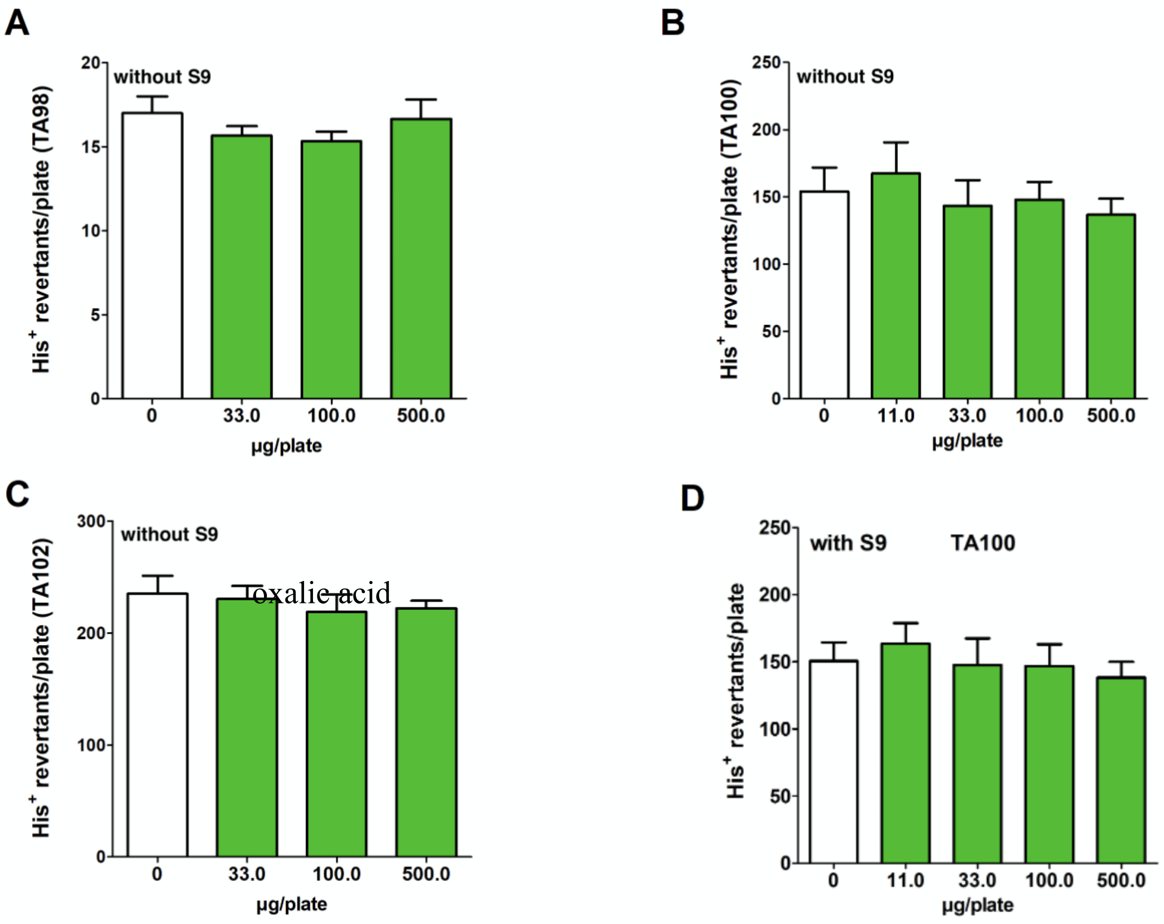

Figure S5: results of a representative experiment reporting mutagenic activities dibromomethanesulfonic acid with Salmonella strains TA98 (A), TA100 with (B) and TA102 (C) without metabolic activation, as well as TA100 with metabolic activation (D). Columns indicate mean ± SD from three plates. * positive according to the two-fold rule. Individual values in Table S8

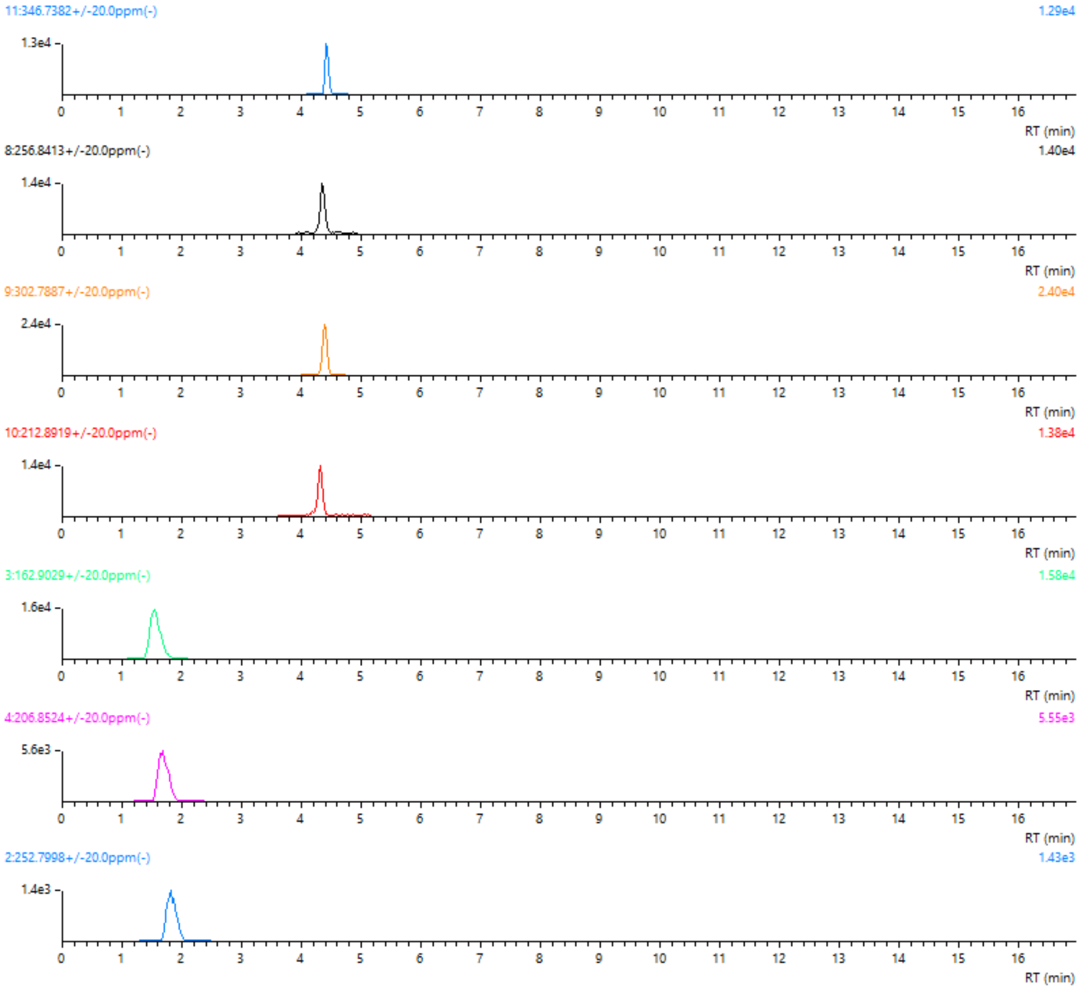

Figure S6: Extracted ion chromatograms of analytes in an unspiked London tap water sample (from top to bottom): 2,2,4-tribromo-5-hydroxycyclopent-4-ene-1,3-dione (t_R= 4.4 min), 2-bromo-2,4-dichloro-5-hydroxycyclopent-4-ene-1,3-dione (t_R=4.3 min), 2,4-dibromo-2-chloro or 2,2-dibromo-4-chloro-5-hydroxycyclopent-4-ene-1,3-dione (t_R= 4.4 min), 2,2,4-trichloro-5-hydroxycyclopent-4-ene-1,3-dione (TCHCD, t_R=4.3 min), dichloromethanesulfonicacid (t_R=1.6 min) and bromochloromethanesulfonic acid (t_R=1.7 min) and dibromomethanesulfonic acid (t_R=1.8 min).

Supplementary Tables

Table S1: Instrumental method details

Instrument	Shimadzu LC-MS 9030 LC-QTOF, with Nexera XR LC system and ESI source	
Column	Waters Atlantis Premier BEH C18 AX (2.5 mm, 2.1x100mm) with pre-column containing identical stationary phase (VanGuard FIT Cartridge, 2.5 µm, 2.1x5 mm)	
Mobile Phase A	5 mM ammonium hydrogen carbonate adjusted to pH 6.9 with acetic acid in water	
Mobile Phase B	5 mM ammonium hydrogen carbonate adjusted to pH 8.9 with diethylamine in water:acetonitrile 1:9	
Flow	0.4 mL/min	
Gradient	Time (min)	%B
	0	5
	1.1	5
	5	50
	5.5	100
	7.25	100
	7.3	5
Total run time	9.3 min	
Injection volume	50 µL	
Injection speed	1 µL/sec	
Interface Voltage	-1 kV	
Nebulizing gas flow	3.0 L/min	
Heating Gas Flow	3.0 L/min	
Interface Temperature	250 °C	
Drying gas flow	10.0 L/min	
DL Temperature	150 °C	
Heat Block Temperature	350 °C	
Mass Calibration	External with NaI	

MS: all events duration 100 ms				
Event (all events duration is 100 ms	scan starts at m/z	scan starts at m/z	Event RT starts (min)	Event RT ends (min)
Full Scan	50.0000	920.0000	0.999	6
Dibromomethanesulfonic acid	252.7498	252.8498	1	2.6
Dichlorometane sulfonic acid	162.8529	162.9529	1	2.6
Bromochloromethanesulfonic acid	206.8024	206.9024	1	2.6
4-bromo-5-hydroxycyclopent-4-ene-1,2,3-trione	202.8485	202.9485	1	2.6
Dibromo-HCD	268.7777	268.8777	2.2	4
Bromo-dichloro-HCD	256.7913	256.8913	3.6	6
Chloro-dibromo-HCD	302.7387	302.8387	3.6	6
Trichloro-HCD	212.8419	212.9419	3.6	6
Tribromo-HCD	346.6882	346.7882	3.8	6
Targeted MS/MS (for confirmation only)				
Product Ion Scan with precurson ion and scanning set for target parent; Collision Energy ramp 35 ± 15 V, events time 150 ms, Q1 Unit Resolution				
Event	Precursor ion	Event RT starts (min)	Event RT ends (min)	
Dibromomethanesulfonic acid	250.8019	1	2.6	
Dichlorometane sulfonic acid	162.9029	1	2.6	
Bromochloromethanesulfonic acid	206.8524	1	2.6	
4-bromo-5-hydroxycyclopent-4-ene-1,2,3-trione	202.8485	1	2.6	
Dibromo-HCD	266.8298	2.2	4	
Bromo-dichloro-HCD	256.8413	3.6	6	
Chloro-dibromo-HCD	300.7908	3.6	6	
Trichloro-HCD	212.8919	3.6	6	
Tribromo-HCD	344.7403	3.8	6	

Table S2: drinking water sampling locations (London Boroughs) and dates.

House Number	Borough	Sampling dates
1	Brent	14/08/2023
		30/08/2023
		12/09/2023
2	Kensington and Chelsea	14/08/2023
		31/08/2023
		12/09/2023
3	Islington	15/08/2023
		30/08/2023
		13/09/2023
4	Wandsworth	15/08/2023
		31/08/2023
		12/09/2023
5	Ealing	15/08/2023
		31/08/2023
		12/09/2023
6	Kensington and Chelsea	15/08/2023
		30/08/2023
		12/09/2023
7	Wandsworth	15/08/2023
		31/08/2023
		13/09/2023

Table S3: 2,2,4-tribromo-5-hydroxycyclopent-4-ene-1,3-dione (TBHCD,) and dibromomethanesulfonic acid (DBMSA) concentrations quantified in London drinking water samples, followed by semi quantitative results for 2-bromo-2,4-dichloro-5-hydroxycyclopent-4-ene-1,3-dione (BDCHCD) (E), 2,4-dibromo-2-chloro or 2,2-dibromo-4-chloro-5-hydroxycyclopent-4-ene-1,3-dione (CDBHCD), 2,2,4-trichloro-5-hydroxycyclopent-4-ene-1,3-dione (TCHCD), dichloromethanesulfonicacid (DCMSA) and bromochloromethanesulfonic acid (BCMSA). Tap water from seven homes were sampled biweekly three times.

House Number	TBHCD	DBMSA	BDCHCD (semi-quantitative)	CDBHCD (semi-quantitative)	TCHCD (semi-quantitative)	DCMSA (semi-quantitative)	BCMSA (semi-quantitative)
1	49	116	78	122	76	1375	488
	166	391	204	416	191	3017	1195
	137	246	163	331	113	2089	881
2	141	777	183	359	163	7152	2507
	136	409	142	308	141	2964	1227
	160	435	119	314	90	2528	1192
3	148	409	145	305	170	1724	640
	130	321	118	214	116	2480	980
	154	395	136	271	90	3159	1268
4	83	152	182	261	231	3305	967
	71	269	123	197	157	2483	931
	62	293	74	142	66	1758	846
5	139	285	198	330	190	4250	1444
	127	226	167	323	146	2196	873
	135	243	172	346	107	2018	861
6	130	586	172	343	144	10727	3200
	134	356	147	315	143	2997	1192
	138	361	128	295	88	2511	1114
7	108	65	184	269	184	2391	626
	65	260	136	193	169	2821	1039
	139	240	172	349	133	2494	976
**Average**	**122**	**326**	**150**	**286**	**138**	**3164**	**1164**

Table S4: 24 and 48 hours stability study in tap and ultrapure water (n=3, duplicate injection). Analyte degradation was expressed as average area percent decrease in comparison to time zero. The % relative standard deviation of Area% over all injections for each condition tested is also reported.

Sample	Storage Condition	Timepoint	TBHCD Area% Decrease in comparison to T0	TBHD Area% %RSD
Municipal water, spiked at 500 ng L^{-1}	-18°C	24 hours	No statistically significant difference with reference	3
	-18°C	48 hours	-13	3
	4°C	24 hours	-12	1
	4°C	48 hours	-39	5
	Room Temperature	24 hours	-85	4
	Room Temperature	48 hours	-98	11
Ultrapure water, spiked at 500 ng L^{-1}	4°C	24 hours	No statistically significant difference with reference	1
	4°C	48 hours	-5	2

Table S5: Bond dissociation enthalpies and energies for n-bromosuccinimide and halogenated hydroxycyclopentenediones calculated in gas phase and water.

	Bond dissociation enthalphy (kcal mol^{-1})		Bond dissociation free energy (kcal mol^{-1})	
**Compound**	**Gas-phase**	**Water**	**Gas-phase**	**Water**
N-bromosuccinimide	67.2	66.6	57.4	56.4
2,2,4-tribromo-5-hydroxycyclopent-4-ene-1,3-dione	52.7	50.6	41.7	39.5
2,4-dibromo-2-chloro-5-hydroxycyclopent-4-ene-1,3-dione (C-Br)	52.5	50	41.5	39.2
2,4-dibromo-2-chloro-5-hydroxycyclopent-4-ene-1,3-dione (C-Cl)	65.2	63.3	54.1	52
2-bromo-2,4-dichloro-5-hydroxycyclopent-4-ene-1,3-dione (C-Br)	52.3	50	41.4	39.1
2-bromo-2,4-dichloro-5-hydroxycyclopent-4-ene-1,3-dione (C-Cl)	64.9	62.9	53.8	51.8
2,2,4-trichloro-5-hydroxycyclopent-4-ene-1,3-dione	64.9	62.4	53.8	51.4

Table S6: Photodegradation of tribromo-HCD. Average area percent variations (n=3) during exposure to sunlight for 2,2,4-tribromo-5-hydroxycyclopent-4-ene-1,3-dione (TBHCD), 2,4-dibromo-5-hydroxycyclopent-4-ene-1,3-dione (DBHCD), 4-bromo-5-hydroxycyclopent-4-ene-1,2,3-trione (BHCT). Areas were measured in a solution 1000 ng L^{-1} of tribromo-HCD in ultrapure water at various intervals of exposure to laboratory light. A% variations calculated as a ratio with TBHCD average area at T0. RH represents methanol in this case, and by extension dissolved organic matter in drinking water.

**Minutes of exposure to natural light**	**TBHCD, Area% of T0**	**DBHCD, Area% formed since T0**	**BHTD, Area% formed since T0**
0	100	0	0
15	93	5	1
30	90	7	1
45	85	10	2
60	81	12	2
75	81	11	2
90	73	17	2

Table S7: pH-induced degradation of 2,2,4-tribromo-5-hydroxycyclopent-4-ene-1,3-dione. Average area percent variations (n=3) for 2,2,4-tribromo-5-hydroxycyclopent-4-ene-1,3-dione (TBHCD), 2,4-dibromo-5-hydroxycyclopent-4-ene-1,3-dione (DBHCD), 4-bromo-5-hydroxycyclopent-4-ene-1,2,3-trione (BHCT) after dilution at 1000 ng L^{-1} in ultrapure water at pH 4, 7.4, 8.5 and 9. Solutions were stored in the dark at room temperature for 1 and 2 hours, each condition tested in triplicate. A% calculated as a ratio with TBHCD average area at T0.

**pH of the solution**	**Storage time (h)**	**TBHCD, Area% of T0**	**DBHCD, Area% formed since T0**	**BHTD, Area% formed since T0**
4	1	no statistically significant difference with T0	no statistically significant difference with T0	no statistically significant difference with T0
4	2	no statistically significant difference with T0	no statistically significant difference with T0	no statistically significant difference with T0
7.4	1	no statistically significant difference with T0	no statistically significant difference with T0	no statistically significant difference with T0
7.4	2	no statistically significant difference with T0	no statistically significant difference with T0	no statistically significant difference with T0
8.5	1	91	4	1
8.5	2	81	5	1
9	1	73	6	2
9	2	58	8	3

Table S8: individual datapoints for Salmonella/microsome assay for 2,2,4-tribromo-5-hydroxycyclopent-4-ene-1,3-dione (A) and dibromomethanesulfonic acid (B)

A:

**Strain**	**TA98 without S9**				
**µg/plate**	**0**	**33.0**	**100.0**	**500.0**	
His+ revertants/plate	18	28	89	4	
His+ revertants/plate	16	29	131	8	
His+ revertants/plate	17	34	96	3	
**Strain**	**TA100 without S9**				
**µg/plate**	**0**	**11.0**	**33.0**	**100.0**	**500.0**
His+ revertants/plate	134	223	470	964	0
His+ revertants/plate	160	220	416	1054	0
His+ revertants/plate	168	218	423	1048	0
**Strain**	**TA102 without S9**				
**µg/plate**	**0**	**33.0**	**100.0**	**500.0**	
His+ revertants/plate	250	310	520	15	
His+ revertants/plate	238	340	522	7	
His+ revertants/plate	218	327	502	8	
**Strain**	**TA100 with S9**				
**µg/plate**	**0**	**11.0**	**33.0**	**100.0**	**500.0**
His+ revertants/plate	171	176	170	540	406
His+ revertants/plate	143	141	156	500	426
His+ revertants/plate	145	176	157	660	411

B:

**Strain**	**TA98 without S9**				
**µg/plate**	**0**	**33.0**	**100.0**	**500.0**	
His+ revertants/plate	18	16	15	16	
His+ revertants/plate	16	15	16	18	
His+ revertants/plate	17	16	15	16	
**Strain**	**TA100 without S9**				
**µg/plate**	**0**	**11.0**	**33.0**	**100.0**	**500.0**
His+ revertants/plate	134	157	129	133	142
His+ revertants/plate	160	194	136	154	123
His+ revertants/plate	168	151	165	157	145
**Strain**	**TA102 without S9**				
**µg/plate**	**0**	**33.0**	**100.0**	**500.0**	
His+ revertants/plate	250	224	220	224	
His+ revertants/plate	218	244	234	228	
His+ revertants/plate	238	224	203	215	
**Strain**	**TA100 with S9**				
**µg/plate**	**0**	**11.0**	**33.0**	**100.0**	**500.0**
His+ revertants/plate	162	180	152	134	144
His+ revertants/plate	135	161	165	142	146
His+ revertants/plate	155	150	126	165	125
